# Manganese Enhances the Osteogenic Effect of Silicon‐Hydroxyapatite Nanowires by Targeting T Lymphocyte Polarization

**DOI:** 10.1002/advs.202305890

**Published:** 2023-12-01

**Authors:** Ruomei Li, Zhiyu Zhu, Bolin Zhang, Ting Jiang, Cheng Zhu, Peng Mei, Yu Jin, Ruiqing Wang, Yixin Li, Weiming Guo, Chengxiao Liu, Lunguo Xia, Bing Fang

**Affiliations:** ^1^ Department of Orthodontics Shanghai Ninth People's Hospital Shanghai Jiao Tong University School of Medicine Shanghai Jiao Tong University 500 Quxi Road Shanghai 200011 China; ^2^ Department of Stomatology XinHua Hospital Affiliated to Shanghai Jiao Tong University School of Medicine Shanghai Jiao Tong University 1665 Kongjiang Road Shanghai 200092 China

**Keywords:** bone regeneration, hydroxyapatite nanowires, manganese, manganese superoxide dismutase, silicon, T lymphocytes

## Abstract

Biomaterials encounter considerable challenges in extensive bone defect regeneration. The amelioration of outcomes may be attainable through the orchestrated modulation of both innate and adaptive immunity. Silicon‐hydroxyapatite, for instance, which solely focuses on regulating innate immunity, is inadequate for long‐term bone regeneration. Herein, extra manganese (Mn)‐doping is utilized for enhancing the osteogenic ability by mediating adaptive immunity. Intriguingly, Mn‐doping engenders heightened recruitment of CD4^+^ T cells to the bone defect site, concurrently manifesting escalated T helper (Th) 2 polarization and an abatement in Th1 cell polarization. This consequential immune milieu yields a collaborative elevation of interleukin 4, secreted by Th2 cells, coupled with attenuated interferon gamma, secreted by Th1 cells. This orchestrated interplay distinctly fosters the osteogenesis of bone marrow stromal cells and effectuates consequential regeneration of the mandibular bone defect. The modulatory mechanism of Th1/Th2 balance lies primarily in the indispensable role of manganese superoxide dismutase (MnSOD) and the phosphorylation of adenosine 5′‐monophosphate‐activated protein kinase (AMPK). In conclusion, this study highlights the transformative potential of Mn‐doping in amplifying the osteogenic efficacy of silicon‐hydroxyapatite nanowires by regulating T cell‐mediated adaptive immunity via the MnSOD/AMPK pathway, thereby creating an anti‐inflammatory milieu favorable for bone regeneration.

## Graphical abstract

In the context of bone healing, the introduction of manganese‐doped silicon‐hydroxyapatite nanowires (Mn‐SiHANWs) induces T helper (Th) 2 cells while concurrently inhibiting Th1 cell polarization. Mechanistically, the presence of Mn‐SiHANWs activates manganese superoxide dismutase (MnSOD) and leads to the phosphorylation of adenosine 5′‐monophosphate‐activated protein kinase (AMPK). This anti‐inflammatory environment, characterized by elevated interleukin (IL)‐4 and decreased interferon gamma (IFN‐γ), ultimately promotes bone marrow stromal cell (BMSC) osteogenesis.

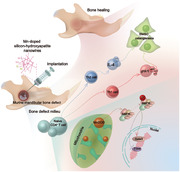

## 1 Introduction

Bone defect regeneration is crucial for restoring craniofacial function and aesthetics.^[^ ^1^ ^]^ Clinical evidence underscores that natural healing of large bone defects is limited to 10% of the defect area, necessitating recourse to autologous bone grafting or biomaterial implantation.^[^ ^2^ ^]^ While traditional biomaterials, such as calcium phosphate bioceramics, are widely used in bone repair due to their excellent biocompatibility, their limited bioactivity remains constrained in meeting the demand for mass bone osteogenesis and functional reconstruction.^[^ ^3^ ^]^ The paradigm of “osteoimmunology,” as introduced by Arron and Choi in 2000, introduces novel strategies for orchestrating osteogenesis and, consequently, effective bone regeneration.^[^ ^4^ ^]^ In this evolving landscape, biomaterials embrace a new role as pivotal regulators of osteoimmunology in bone healing, ushering forth intrinsic inflammatory responses upon implantation.^[^ ^5^ ^]^ The cascade of local inflammation commences with the recruitment of neutrophils, monocytes, and macrophages—the vanguards of innate immunity—succeeded by a protracted lymphocyte infiltration, namely T and B cells, emblematic of adaptive immunity.^[^ ^6^ ^]^ Our previously established (T cell receptor β chain‐deficient) Tcrβ*^−/−^* transgenic mouse model confirmed that T cell immunodeficiency inhibited bone repair, which provided compelling evidence that T cells are critical for bone regeneration.^[^ ^6^ ^]^ Despite prevailing biomaterial interventions predilection for innate immune system modulation, their influence remains confined therein which compels the exploration of avenues to extend their purview to the adaptive immune domain. We have previously undertaken silicon (Si) doping to enhance angiogenesis and osteogenesis within nanosized hydroxyapatite.^[^ ^7^ ^]^ Notably, Si, during the early inflammatory phase post‐implantation, exhibits immunoregulatory properties that inhibit macrophage‐mediated pro‐inflammatory responses^[^ ^8^ ^]^ while activating monocytes.^[^ ^9^ ^]^ In addressing the exigency for substantial bone regeneration, we postulate that the immunoregulatory and osteogenic effects of Si‐hydroxyapatite could be enhanced through the infusion of novel elements capable of finely regulating T cell dynamics during bone healing.

The intrinsic modulation of infiltrating T cells assumes a pivotal role in both pro‐ and anti‐inflammatory tenets through polarization into distinct subsets. The secretion of interferon gamma (IFN‐γ), interleukin (IL)−12, and tumor necrosis factor (TNF)‐α secreted by T helper (Th) 1 cells orchestrates macrophage‐mediated infection resistance, osteoclastogenesis induction, and fibrogenesis promotion.^[^ ^10^ ^]^ Conversely, Th2 cells secrete IL‐4, IL‐10, and IL‐13, which prevent bone destruction and promote bone healing.^[^ ^11^ ^]^ Therefore, creating an immune microenvironment that eliminates Th1 while promoting Th2 polarization at the site of the defect may block the pro‐inflammatory reaction and promote osteogenesis.^[^ ^10^, ^11^, ^12^ ^]^

The equilibrium of Th1/Th2 dynamics is subject to the sway of multifaceted determinants.^[^ ^13^ ^]^ Peptide dosage, T cell receptor (TCR) affinity,^[^ ^14^ ^]^ energy metabolism (e.g., oxidative phosphorylation [OXPHOS], glycolysis, and fatty acid metabolism),^[^ ^15^ ^]^ as well as lysosomal functionality, constitute a subset of these influential factors. OXPHOS exerted a negative regulatory effect on Th1‐related functions, while the inhibition of Th2 responses in an allergic asthma model is observed as a result of abnormalities in OXPHOS.^[^ ^16^ ^]^ The metabolic processes of glutamine and glycolysis have been found to facilitate the synthesis of IFN‐γ by Th1 cells.^[^ ^17^ ^]^ The augmentation of Th2 cell responses is facilitated by the accumulation of adenosine triphosphate (ATP) and the process of fatty acid oxidation.^[^ ^18^ ^]^ Manganese superoxide dismutase (MnSOD), stationed within mitochondria, assumes a cardinal role in energy metabolism. Previous studies have reported that MnSOD deficiency leads to defective T cell development and apoptosis.^[^ ^19^ ^]^ MnSOD decreases hyperoxia‐induced IL‐8 production, curbing hyperoxic lung injury. Additionally, it curtails lung inflammation by hampering the secretion of pro‐inflammatory cytokines, including TNF‐α, IL‐1β, and IL‐6, while concurrently promoting the secretion of the anti‐inflammatory cytokine IL‐10.^[^ ^20^ ^]^ It is reasonable to assume that MnSOD serves as a potential mediator of Th1/Th2 balance within the purview of energy metabolic regulation.

As a trajectory to establish a MnSOD‐mediated anti‐inflammatory environment, we intended to introduce manganese (Mn) to the regenerative milieu. The Mn ion operates within the enzyme active center, thereby exerting control over the enzymatic activity of MnSOD.^[^ ^21^ ^]^ The integration of Mn ions into Si‐hydroxyapatite nanowires emerges as a proposition to magnify their immunoregulatory and osteogenic effects for mass bone regeneration. This undertaking, however, hinges upon the strategic manipulation of T lymphocyte‐mediated adaptive immunity via Mn‐doping. In this study, animal models and cell cultures collectively indicated that Mn‐doping environments stimulate a shift from Th1 to Th2 polarization. Then, a series of experiments were conducted to explain how this biological process promotes osteogenesis. The underlying mechanism by which Mn‐doping manipulates T lymphocyte polarization has been unraveled, thereby enriching our understanding of the importance of Mn in adaptive immunity and simultaneously charting the theoretical terrain for harnessing T lymphocyte polarization in bone regeneration. Clinically, this study may provide insights concerning the incorporation of Mn in traditional biomaterials for extensive immunoregulation in mass bone defects.

## 2 Results and Discussions

### 2.1 Characteristics and Biological Effects of Manganese‐Doped Silicon‐Hydroxyapatite Nanowires

**Figure 1:**
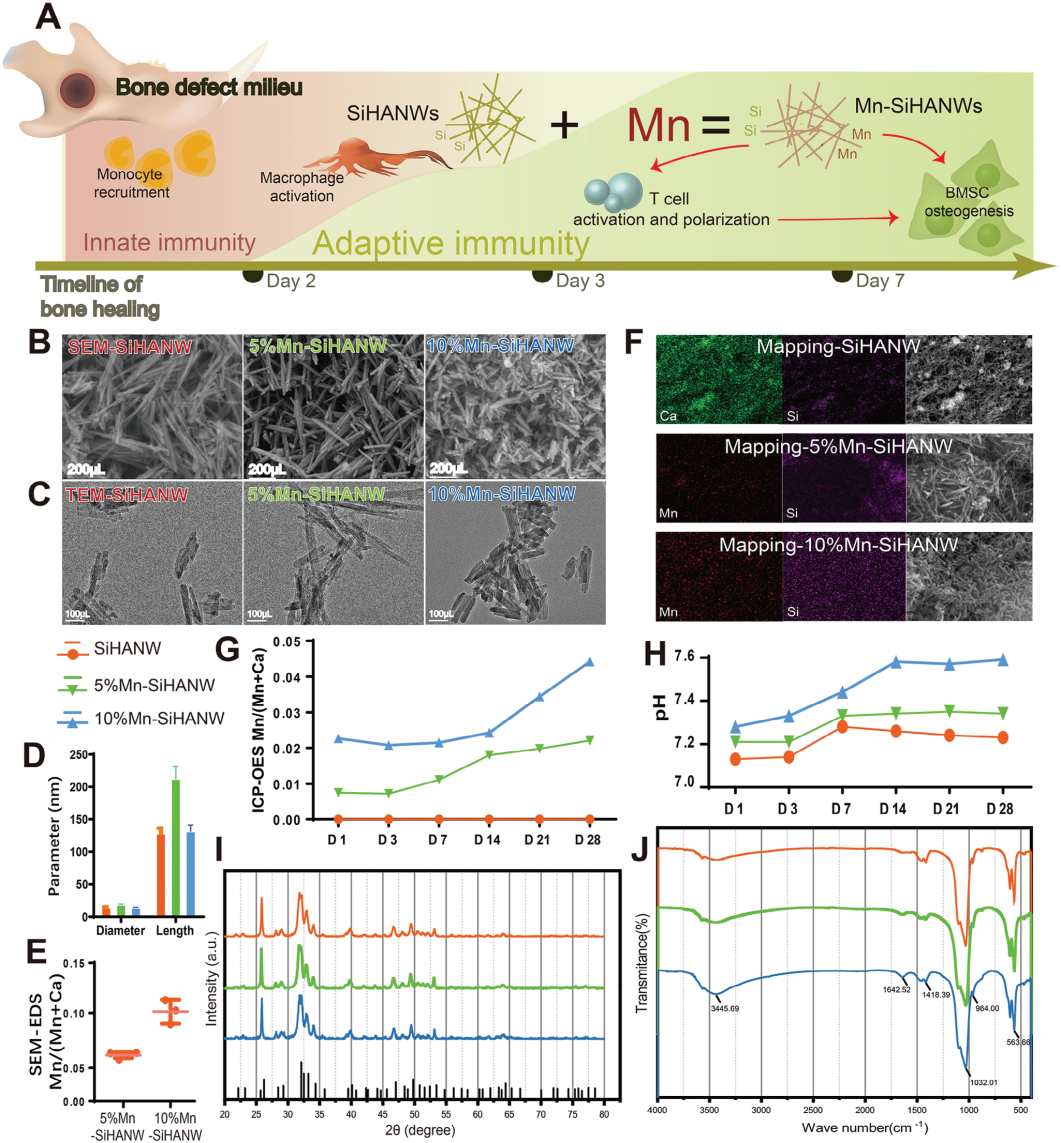
Schematic representation and characteristics of silicon‐hydroxyapatite nanowire (SiHANW), 5% manganese‐doped silicon‐hydroxyapatite nanowire (Mn‐SiHANW), and 10% Mn‐SiHANW. A) An Illustrative diagram depicting the designation and function of silicon‐hydroxyapatite nanowires doped with or without manganese. B) Scanning electron microscope (SEM). C) Transmission electron microscope (TEM). D) Key parameters of SiHANW, 5% Mn‐SiHANW, and 10% Mn‐SiHANW. E) Scanning electron microscopy‐energy dispersive spectroscopy (SEM‐EDS) quantification of manganese (Mn)/(Calcium (Ca)+Mn) mole ratios. F) Elemental mapping of Ca, Mn, and silicon (Si). G) Inductively coupled plasma optical emission spectroscopy (ICP‐OES) assessment of Mn/(Ca+Mn) molar ratios. H) Pondus hydrogenii (pH) measurements in phosphate‐buffered saline (PBS) supplemented with SiHANW, 5% Mn‐SiHANW, and 10% Mn‐SiHANW. I) X‐ray diffraction (XRD) patterns. J) Fourier transform infrared spectroscopy (FTIR) spectra of SiHANW, 5% Mn‐SiHANW, and 10% Mn‐SiHANW.

Herein, manganese has been successfully doped into silicon‐hydroxyapatite nanowires (Mn‐SiHANWs), which is supposed to expand the immunomodulatory effect of silicon‐hydroxyapatite nanowires (SiHANWs) from macrophage‐mediated innate immunity to T cell‐mediated adaptive immunity (**Figure** **1**A). Different Mn/(Calcium (Ca)+Mn) mole ratios (0.05 and 0.1) of Mn‐SiHANWs (5% Mn‐SiHANWs and 10% Mn‐SiHANWs) were synthesized by hydrothermal reaction. Synchronously, SiHANWs were fabricated as a control. Scanning electron microscope (SEM) and transmission electron microscope (TEM) micrographs uniformly presented short‐wire–like morphologies (Figure 1B,C). The mean diameter ≈15 nm (Figure 1D and Table S1, Supporting Information) (SiHANWs, 14.35 ± 2.92 nm; 5% Mn‐SiHANWs, 19.34 ± 3.95 nm; 10% Mn‐SiHANWs, 13.98 ± 3.75 nm) resonates with hydroxyapatite nanocrystals encountered within bone tissue.^[^ ^22^ ^]^ The lengths range from 50.33 to 449.44 nm (Figure 1D and Table S1, Supporting Information) (Mn‐SiHANWs, 50.33 to 212.52 nm; 5% Mn‐SiHANWs, 52.57 to 449.44 nm; 10% Mn‐SiHANWs, 67.14 to 204.16 nm).

The Mn/(Ca+Mn) mole ratios were 0.067 ± 0.002 and 0.101 ± 0.009, tested and calculated by scanning electron microscopy‐energy dispersive spectroscopy (SEM‐EDS) (Figure 1E and Table S2, Supporting Information). According to the elemental mapping, Mn disperses in 5% Mn‐SiHANWs and 10% Mn‐SiHANWs, whereas Si is homogeneously distributed in all groups (Figure 1F).

The supernatants of 5% Mn‐SiHANWs or 10% Mn‐SiHANWs dissolved in phosphate‐buffered saline (PBS) over durations of 1, 3, 7, 14, 21, and 28 days have been assessed through inductively coupled plasma optical emission spectroscopy (ICP‐OES). The calculated Mn/(Ca+Mn) ratios rose from 0.005 to 0.022 and from 0.017 to 0.033 for 5% Mn‐SiHANWs and 10% Mn‐SiHANWs, respectively (Figure 1G and Table S3, Supporting Information). Concomitantly, pondus hydrogenii (pH) values ranged from 7.11 to 7.59 (Figure 1H). Subsequent X‐ray diffraction (XRD) profiles meticulously align with hydroxyapatite crystal structures of SiHANWs, 5% Mn‐SiHANWs, and 10% Mn‐SiHANWs, validating their structural congruence (Figure 1I, JCPDS card No. 09–0432). Fourier transform infrared spectroscopy (FTIR) spectra were identified according to the parameters of hydroxyapatite. Noteworthy peaks at 563.66, 964, and 1032.01 cm^−1^ demonstrate the presence of PO_4_^3−^. Characteristic peaks at 3445.69 cm^−1^ resonate with the OH group (Figure 1J).

The strategic determination of an optimal ion‐doped biomaterial concentration assumes pivotal significance to preclude undesirable side effects caused by overdosage while maintaining osteogenic efficacy.^[^ ^23^ ^]^ In pursuit of this, the concentrations from 0 to 100 µg mL^−1^ of SiHANWs, 5% Mn‐SiHANWs, and 10% Mn‐SiHANWs were analyzed. According to the immunostainings, bone marrow stromal cells (BMSCs) retained normal morphologies after co‐culturing with 0.1 and 1 µg mL^−1^ suspending liquid of SiHANWs, 5% Mn‐SiHANWs, and 10% Mn‐SiHANWs (Figure S1A, Supporting Information). Corresponding cell counting kit‐8 (CCK‐8) assays supported these findings and depicted mild toxicity in 10 and 100 µg mL^−1^ suspending liquid, especially for 10% Mn‐SiHANWs (Figure S1A, Supporting Information). Further substantiation is presented through safranin‐O staining, elucidating the mineralization induced by 1 µg mL^−1^ SiHANWs, 5% Mn‐SiHANWs, and 10% Mn‐SiHANWs in BMSC (Figure S1B,C, Supporting Information). Noteworthy elevations were evident in osteonectin (Figure S1E, Supporting Information) in the 5% Mn‐SiHANW group. Runt‐relate transcription factor 2 (Runx2) increased in the SiHANW, 5% Mn‐SiHANW, and 10% Mn‐SiHANW groups (Figure S1D, Supporting Information). Equally, osteocalcin (OCN) (Figure S1F, Supporting Information) registers marked augmentation within the precincts of the 5% Mn‐SiHANW and 10% Mn‐SiHANW groups.

The bone‐mimicking nanosized structure affords hydroxyapatite a potent osteogenic ability by promoting BMSC stretching, cellular adhesion, cell‐extracellular matrix interactions, cell proliferation, and osteogenesis, surmounting the capabilities of analogous micron‐sized particles of the same composition.^[^ ^24^ ^]^ The fortification of hydroxyapatite with bioactive ions (including zinc (Zn), magnesium, Mn, copper, strontium, and Si) confers multiple osteogenic‐related functions spanning mechanical properties, antibacterial ability, and osteoimmunomodulatory potency.^[^ ^23^, ^25^ ^]^ Mn can improve the mechanical properties of hydroxyapatite, promote osteoblast proliferation and mineralization, and inhibit osteoclastic effects.^[^ ^26^ ^]^

The Mn‐SiHANWs synthesized in this study exhibit a bone‐mimicking nanowire structure akin to SiHANWs. Intriguingly, certain Mn‐doping concentrations stimulated prominent proliferation, osteogenic, and mineralization effects within BMSC in vitro. These effects coincide with those of other Mn‐doped hydroxyapatite materials in bone regeneration.^[^ ^27^ ^]^

### 2.2 Facilitation of Murine Mandibular Bone Healing by Mn‐SiHANW

The divergences between in vitro and in vivo osteogenic responses confer formidable challenges upon biomaterial engineering, especially within ion‐doped biomaterials.^[^ ^24^, ^28^ ^]^ In order to assess the osteogenic effects of SiHANWs, 5% Mn‐SiHANWs, and 10% Mn‐SiHANWs in vivo, they were dispersed in Gelma hydrogels (Gelma), resulting in the formulations Gelma+SiHANW, Gelma+5%Mn‐SiHANW, and Gelma+10%Mn‐SiHANW, with Gelma serving as the control. Subsequent administration of these formulations involved their injection into the mandibular bone defects, undertaken in accordance with a standardized procedure that was described in our previous study.^[^ ^29^ ^]^

The injectability, SEM, and mechanical properties are displayed in Figure S2, Supporting Information. The great injectability of Gelma does not change with the presence of Mn‐SiHANW (Figure S2A, Supporting Information). The mechanical properties were analyzed by dynamic sweep frequency rheological studies (Figure S2B, Supporting Information). From 0.1 to 10 Hz, the storage modulus (G′) surpassed the loss modulus (G″), indicating that all groups of hydrogels possess satisfying stability and elastic solids (Figure S2B, Supporting Information). All groups present interconnected porous network structures (Figure S2C, Supporting Information), allowing the infiltration of immunocytes and tissue regeneration.

**Figure 2:**
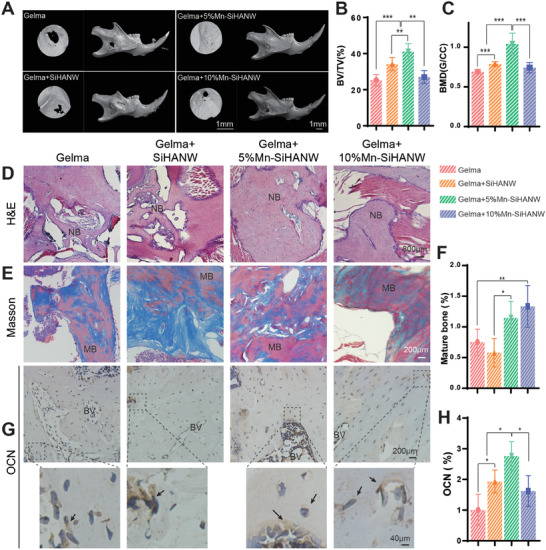
Differential Efficacy of Gelma Hydrogels (Gelma), Gelma+SiHANW, Gelma+5%Mn‐SiHANW, and Gelma+10%Mn‐SiHANW in mandibular bone healing. A) Three dimensional (3D) reconstructed micro‐CT images of mandibles after implantation for 8 weeks. B) Quantitative analysis of bone volume/total tissue volume (BV/TV). C) Quantitative analysis of bone mineral density (BMD). D) Histological evaluation of new bone formation revealed by hematoxylin and eosin (H&E) staining. E) Masson staining and F) quantification of mature bone. G) Immunohistochemical staining and quantification H) of Osteocalcin (OCN), NB, BV, and MB. The arrows denote new bone, blood vessels, mature bone, and osteoblasts. *N* = 5, *P < 0.05, **P < 0.01, ***P < 0.001.

As shown in 2D reconstructions, SiHANWs, 5% Mn‐SiHANWs, and 10% Mn‐SiHANWs strengthened Gelma's ability to promote murine mandibular bone healing. Eight weeks after the surgery, the Gelma+5%Mn‐SiHANW group exhibited substantial bone growth (**Figure** **2**A). Quantitative scrutiny of micro‐computed tomography (micro‐CT) results corroborates these observations, revealing noteworthy amplifications in bone mineral density (BMD, surging by 1.3‐fold compared to the Gelma+SiHANW group) and bone volume/total tissue volume (BV/TV, augmenting by 1.2‐fold compared to the Gelma+SiHANW group) within the Gelma+5%Mn‐SiHANW assembly (Table S4, Supporting Information). Conversely, the Gelma+10%Mn‐SiHANW group exhibited less bone formation compared with the Gelma+5%Mn‐SiHANW and Gelma+SiHANW groups (Figure 2B,C). Histological analysis indicates the emergence of nascent bone across all groups, notably pronounced in the Gelma+SiHANW and Gelma+5%Mn‐SiHANW groups (Figure 2D). Moreover, Gelma+5%Mn‐SiHANW and Gelma+10%Mn‐SiHANW exhibit superior deposition of mature bone within the defect area, with Gelma+SiHANW displaying a comparatively diminished extent of mature bone (Figure 2E,F). Notably, Gelma+5%Mn‐SiHANW induced more OCN expression, emblematic of heightened bone maturation and formation during the latter stages of osteogenesis (Figure 2G,H).

These findings allude to the augmentation of bone filling within the defect region upon judicious Mn concentration. Intriguingly, the unsatisfactory osteogenic outcome associated with 10% Mn‐SiHANW could be attributed to an over‐release of Mn released during implantation. This proposed a much more sensitive reaction in murine mandibular bone than that in BMSC in vitro. Thus, 10% Mn‐SiHANW was eliminated from subsequent experimental groups.

### 2.3 Mn‐SiHANWs Assist Bone Healing by Increasing Th2 Cell Polarization While Decreasing Th1 Cell Polarization

**Figure 3:**
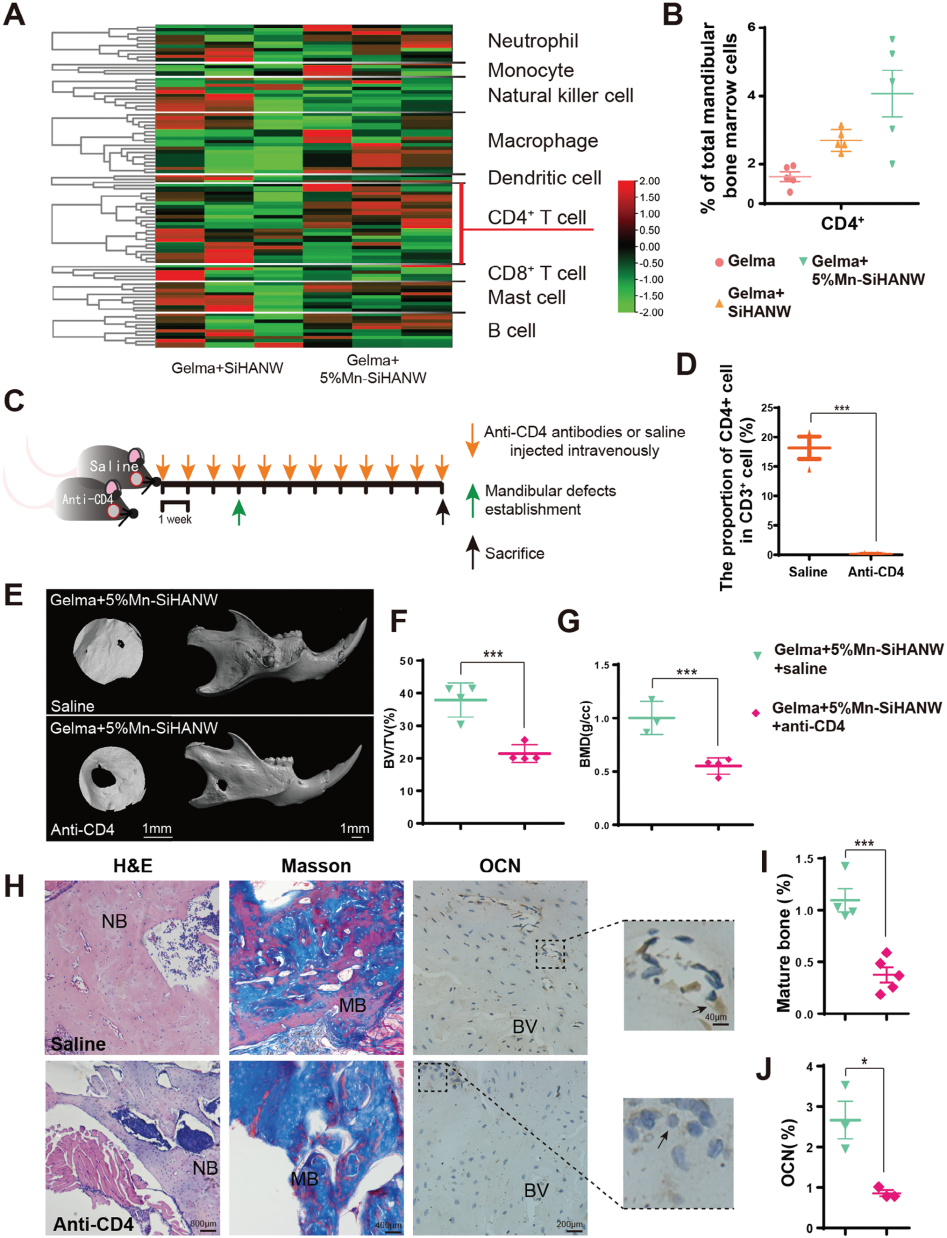
Polarization of local and systemic T cells after implantation of Gelma, Gelma+SiHANW, and Gelma+5%Mn‐SiHANW into the mandibular defects. A) Heatmap illustrating gene expression profiles of immune cells surrounding wound areas treated with Gelma+SiHANW or Gelma+5%Mn‐SiHANW. B) CD4^+^ T cell proportion in murine mandibular bone defects on day 4. C) Schematic representation of generating mandibular bone defects in CD4^+^ T cell‐depleted and control mice via intermittent treatment of anti‐CD4 antibodies and saline intravenously, respectively, followed by mandibular defect establishment. D) Detection of CD4^+^ T cell depletion efficiency. CD4^+^ T cells were undetectable after treatment with anti‐CD4 antibodies. E) The osteogenic abilities of control and CD4^+^ T cell‐depleted murine mandibular defects treated with Gelma+5%Mn‐SiHANW analyzed by three constructed images. F) BV/TV. G) BMD. H) Histological assessment of bone formation using H&E, Masson, and OCN staining. I) Quantification of mature bone using Masson staining. J) Quantification of OCN. NB: New bone; BV: Blood vessel; MB: Mature bone; Arrow: Osteoblast. *N* ≥ 3, *P < 0.05, **P < 0.01, ***P < 0.001.

An exploration into the pivotal functional types of immune subsets conducive to bone healing in a Mn‐existing environment precipitated an examination of mandibular bone tissue, wherein Gelma+SiHANW and Gelma+5%Mn‐SiHANW were implanted, followed by their subsequent analysis through ribose nucleic acid (RNA)‐sequence methodologies (**Figure** **3**A). Employing hierarchical clustering techniques, the variance in gene expression attributable to the diverse immune cell markers was dissected to discern the specific cell type that evinced the most pronounced alteration in gene expression in response to Mn stimulation (Figure 3A). The heatmap showed that CD4^+^ T cells exhibited the most conspicuous modulation in gene expression, characterized by opposing expression patterns. Notably, flow cytometry analysis corroborated these observations, with CD4^+^ T cells exhibiting more frequent aggregation at the implantation site within the Gelma+5%Mn‐SiHANW assembly (4.068% ± 0.684%) in comparison to the Gelma+SiHANW assembly (2.694% ± 0.142%, Figure 3B). T cell infiltration, particularly T helper cells, augments tissue regeneration. The infiltration of T cells enhanced mandibular bone healing, as shown by Yu et al.^[^ ^6^ ^]^ Furthermore, gut‐primed T cells affect bone health in contexts such as osteoporosis and the bony callus phase of fracture healing.^[^ ^30^ ^]^ We therefore speculated that the abundant infiltration of CD4^+^ T cells may be a crucial factor fostering favorable bone healing within the purview of Gelma+5%Mn‐SiHANW implantation. In addition to CD4^+^ T cells, it is worth noting that neutrophils, macrophages, and B cells may also undergo alterations in response to Mn‐doing. Consequently, future investigations should consider the involvement of additional immune cell types in order to comprehensively capture the entirety of immune reactions.

In vivo CD4^+^ T depletion has been extensively performed to study T helper cell subsets. Recombination activating gene 1‐deficient (Rag1*^−/−^*) and Tcr‐β*^−/−^* transgenic mice were frequently utilized to study immune deficiencies, whereas in our study, CD4 knockout techniques or anti‐CD4 neutralized antibody injections can directly highlight our primary pursuit.^[^ ^31^ ^]^ Generating CD4 knockout mice engenders substantial financial resources and temporal investments, whereas the employment of anti‐CD4 antibodies pertains to immunological techniques that effectuate changes within the immune system and its reactions.^[^ ^32^ ^]^ Consequently, the present study has adopted the use of anti‐CD4 antibodies to establish the indispensability of CD4^+^ T cells during bone repair.

To elucidate the specific contribution of CD4^+^ T cells to the process of bone regeneration, a murine model with depleted CD4^+^ T cells was generated through tail vein injection of anti‐CD4 antibodies (Figure 3C). A parallel group of mice receiving saline injections intravenously served as the control for the CD4^+^ T cell‐depleted groups. Three weeks post‐injection, CD4^+^ T cells were scarcely spotted among the blood cells of CD4^+^ T cell‐depleted mice (saline group: 18.200% ± 3.290%, CD4^+^ T cell‐depleted group: 0.888% ± 0.105%, *P* < 0.001, *n* = 3, Figure 3D). Intriguingly, the percentage of CD8^+^ T cells was moderately enriched following CD4^+^ T cell depletion (saline group: 12.167% ± 1.443%, CD4^+^ T cell depleted group: 19.420% ± 1.502%, *P* < 0.001, *n* = 3, Figure S3, Supporting Information). This may be a compensating reaction, and a similar phenomenon has been seen in the increase of TCR‐γδ population after CD4^+^ or CD8^+^ T cell depletion.^[^ ^33^ ^]^ Previous studies indicated, controversially, that CD8^+^ T cells are associated with impaired cancellous bone repair while inducing bone formation via secreting Wnt10b in osteoporosis.^[^ ^34^ ^]^ Nevertheless, the role of CD8^+^ T cells in Mn‐related bone repair remains ambiguous.

After CD4^+^ T cells were successfully depleted, a circular mandibular defect was made, followed by instant Gelma+5%Mn‐SiHANW implantation. After 8 weeks of recovery, micro‐CT imaging revealed discernibly aggravated bone abnormalities in CD4^+^ T cell‐depleted mice (Figure 3E). This was further corroborated by quantification, indicating a significant reduction in both BMD and BV/TV within the CD4^+^ T cell‐depleted group (Figure 3F,G and Table S5, Supporting Information). Notably, markers of bone formation, including new bone formation, mature bone deposition, and OCN expression, all exhibited a notable decline in the CD4^+^ T cell‐depleted mice (Figure 3H–J). These findings underscore the pivotal role of CD4^+^ T cells in mandibular bone regeneration. A similar indispensable function of CD4^+^ T cells has been proposed in diverse tissues such as tendons, liver, skin, and lungs, wherein their contributions encompass immune cell recruitment, inflammation control, angiogenesis, matrix formation, and fibrogenic prevention.^[^ ^35^ ^]^ The main subsets of CD4^+^ T cells, including Th1, Th2, Th17, Th22, Treg, and γδ T, manipulate the intrinsic balance of pro‐ and anti‐inflammatory responses via secreting an array of cytokines such as IFN‐γ, TNF‐α, IL‐2, IL‐4, IL‐6, IL‐10, IL‐12, IL‐13, IL‐17, IL‐22, IL‐33, and TGF‐β.

**Figure 4:**
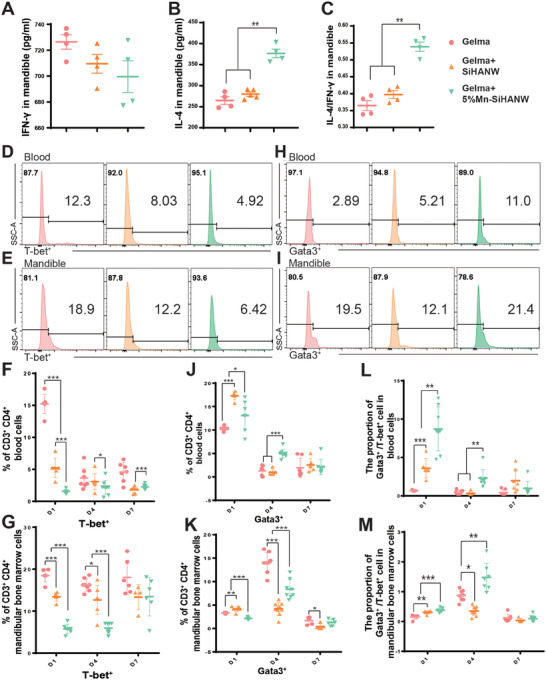
T cell function and polarization post Gelma, Gelma+SiHANW, and Gelma+5%Mn‐SiHANW implantation. A–C) Enzyme‐linked immunosorbent assay (ELISA) quantification of Interferon gamma (IFN‐γ), interleukin (IL)−4, and IL‐4/IFN‐γ ratios 4 days post‐implantation. D) The proportion of T‐bet^+^ cells in the blood and E) mandible 4 days after implantation. F) The proportion of T‐bet^+^ cells in the blood and G) mandible from day 1 to day 7 after implantation. H) The proportion of Gata3^+^ cells in the blood and I) mandible 4 days after implantation. J) The proportion of Gata3^+^ cells in the blood and K) mandible from day 1 to day 7 after implantation. L) The proportion of Gata3^+^/T‐bet^+^ cells in the blood and M) mandible from day 1 to day 7. *N* > 3, *P < 0.05, **P < 0.01, ***P < 0.001.

To delineate the roles of CD4^+^ T cell subsets in the Mn‐existing bone defect milieu, bone tissue from the mandibular defect area implanted with Gelma, Gelma+SiHANW, and Gelma+5%Mn‐SiHANW was collected independently at 1‐, 4‐, and 7 days intervals post‐surgery. The quantification of cytokine production was executed via an enzyme‐linked immunosorbent assay (ELISA). Th2‐derived IL‐4 exhibited heightened expression in the Gelma+5%Mn‐SiHANW group, concomitant with a suppression of Th1‐derived IFN‐γ within both Gelma+SiHANW and Gelma+5%Mn‐SiHANW groups on day 4 (**Figure** **4**A,B). This dynamic shift is further underscored by the discernible augmentation of the IL‐4/IFN‐γ ratio within the defect region implanted with Gelma+5%Mn‐SiHANW (Figure 4C). The T cell polarization was examined by flow cytometry. Gelma+SiHANW and Gelma+5%Mn‐SiHANW restrained Th1 (T‐bet^+^) cell polarization in both the blood and the mandible, with a more pronounced effect observed in the blood on day 1 and in the mandible on days 1 and 4 (Figure 4D–G). Remarkably, on the day immediately following implantation, the Th2 (Gata3^+^) cell population in the Gelma+5%Mn‐SiHANW group appeared diminished relative to the Gelma+SiHANW group (Figure 4K). However, Th2 cells in the Gelma+5%Mn‐SiHANW group gradually accumulated at the defect site and in the circulation, ultimately exceeding the Th2 population in the Gelma+SiHANW group by day 4 (Figure 4H–K). The ratio of Th2/Th1 in the defect area exhibited steady augmentation from days 1 to 4 following Gelma+5%Mn‐SiHANW implantation (Figure 4M), in contrast to the constant ratio within the Gelma or Gelma+SiHANW groups (Figure 4M). Systemically, in blood samples, the ratios of Th2/Th1 rose prominently in both Gelma+SiHANW and Gelma+5%Mn‐SiHANW groups at day 1 (Figure 4L). On day 4, only blood samples from the Gelma+5%Mn‐SiHANW group presented comparatively high Th2/Th1 ratios (Figure 4L). Collectively, both SiHANW and Mn‐SiHANW inhibited the Th1‐related pro‐inflammatory reaction generated by Gelma implantation. Mn‐SiHANW further inhibited the pro‐inflammatory reaction and elevated the Th2‐related anti‐inflammatory reaction compared to SiHANW. Therefore, Mn‐doping can skew the Th1/Th2 balance toward an anti‐inflammatory milieu.

Mn‐SiHANW in this investigation is not the only case that can manipulate the Th1/Th2 balance. The balance turbulences across diverse pathological and physiological contexts. For instance, probiotic‐derived polysaccharide capsules orchestrate a balanced Th1/Th2 response to confer protection against allergy disorders.^[^ ^36^ ^]^ The catalpol‐regulated Th1/Th2 balance alleviates estrogen deficiency‐induced osteoporosis.^[^ ^37^ ^]^ Zn^2+^ signaling alters the Th1/Th2 balance to favor Th1 cell development.^[^ ^38^ ^]^ In parallel, the augmentation in surface roughness and hydrophilicity of titanium implants locally attracts Th2 cells to expedite bone regeneration.^[^ ^39^ ^]^ A porcine tissue‐derived extracellular matrix bio‐scaffold favored muscle regeneration and repair by polarizing Th1 to Th2 through the mTOR/Rictor pathway.^[^ ^31a^ ^]^ However, it is imperative to exercise prudence in over‐inducing Th1‐to‐Th2 polarization, given the potential compromise of the capacity to combat bacterial infections due to the attenuation of Th1‐mediated antimicrobial responses.^[^ ^40^ ^]^ Our objective should encompass the harmonization of Th1 and Th2 functions to advance the ultimate objective of efficacious bone defect healing, rather than merely emphasizing Th2 polarization. Herein, we elaborate on the local and systemic phenotypic and functional changes that Mn‐doping brought to T cells. The functionality of Th2 cells is prominently augmented, while Th1 cell function is suppressed but not entirely eradicated. Further investigations are still required to ascertain whether the antimicrobial potency remains sufficiently robust to address the infected bone defects. Thus, the Mn‐induced antibiotic effect warrants consideration, necessitating an intricate dissection of the interactions amongst pathogenic bacteria, recruited neutrophils, monocytes, inflammatory macrophages, and Th1 cells. The subsequent part of the manuscript will entail an in vitro examination, striving to elucidate the multifactorial components underlying Mn‐induced osteogenesis.

### 2.4 Mn‐SiHANW–Induced Th1/Th2 Balance Affects BMSC Osteogenesis

**Figure 5:**
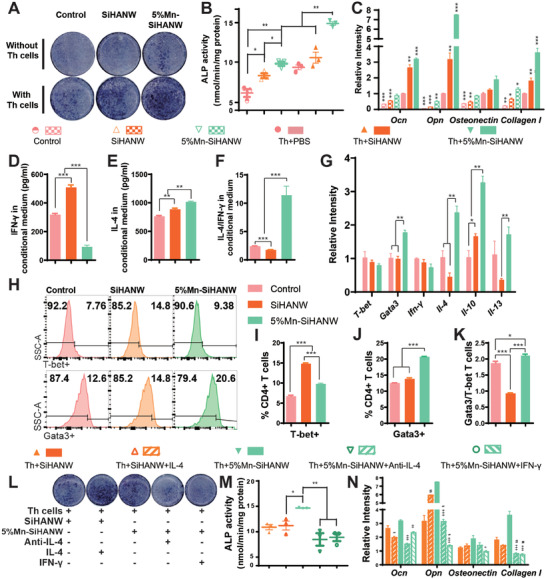
In vitro osteogenic effects and effective cytokine responses induced by Mn‐SiHANW–stimulated T helper (Th) cells. A) Alkaline phosphatase (ALP) staining and B) quantification of bone marrow stromal cells (BMSCs) incubated in conditioned medium derived from PBS (Control), SiHANWs, or 5% Mn‐SiHANWs with or without T helper cells. *P < 0.05, **P < 0.01, ***P < 0.001. C) RT‐PCR analysis of OCN, Osteopontin (Opn), Osteonectin, and Collagen I gene expressions in BMSC stimulated by the conditioned medium. *P < 0.05, **P < 0.01, ***P < 0.001 versus the Th+PBS group. D) IL‐4, E) IFN‐γ, and F) their ratio in conditioned medium of Th+PBS, Th+SiHANWs, or Th+5%SiHANWs. G) Reverse transcription polymerase chain reaction (RT‐PCR) analysis of anti‐ and pro‐inflammatory genes. *P < 0.05, **P < 0.01, ***P < 0.001. H) Flow cytometry analysis of Th1 (T‐bet^+^) and Th2 (Gata3^+^). I) The proportion of Th1, J) Th2, and K) their ratio of CD4^+^ T cells stimulated with PBS, SiHANWs, and 5% Mn‐SiHANWs. *P < 0.05, **P < 0.01, ***P < 0.001. L) ALP staining and M) quantification of BMSC stimulated by the conditioned medium supplemented with IL‐4 recombinant protein, anti‐IL‐4 antibodies, or IFN‐γ recombinant protein. *P < 0.05, **P < 0.01. N) RT‐PCR analysis of OCN, Opn, Osteonectin, and Collagen I gene expressions in BMSC stimulated by the conditioned medium. #*P* < 0.05, ##*P* < 0.01, ###*P* < 0.001 versus the Th+SiHANW group; +*P* < 0.05, ++*P* < 0.01, +++*P* < 0.001 versus the Th+5% Mn‐SiHANW group. *N* = 3.

The pivotal role of T helper cells in the context of Mn‐SiHANW–assisted bone defect healing was underscored through in vivo experiments. To delve deeper into the mechanistic intricacies of the Mn‐imbued osteogenic milieu, we embarked on a series of subsequent investigations. Initially, murine BMSCs were subjected to incubation in conditioned mediums derived from three distinct groups: PBS (Control), SiHANWs, or 5% Mn‐SiHANWs, in combination with or without T helper cells. This study delineated that Mn amplifies the osteogenic potential of SiHANWs, and this effect is further potentiated upon co‐culture with T helper cells (**Figure** **5**A,B). Notably, the osteogenic markers—OCN, osteopontin (Opn), osteonectin, and Collagen I—unfolded a trend wherein T helper cells co‐existing with 5% Mn‐SiHANW (Th+5% Mn‐SiHANW) notably intensified BMSC osteogenesis, particularly in the late stage of osteogenic progression (Figure 5C). Evidently, beyond its direct promotion of osteogenesis, Mn‐SiHANW orchestrates an indirect osteogenic influence via modulation of T helper cell function.

Unveiling the underlying mechanisms governing this Mn‐SiHANW–induced BMSC osteogenesis was pursued by dissecting the effective components within the conditioned medium. Employing an ensemble of techniques encompassing ELISA, reverse transcription polymerase chain reaction (RT‐PCR), and flow cytometry, the Th+5%Mn‐SiHANW group displayed heightened IL‐4 protein and attenuated IFN‐γ protein levels in comparison to the Th+SiHANW group (Figure 5D–F). In sharp contrast, marginal secretion of IFN‐γ and IL‐4 was noted in the Th+PBS group, possibly attributable to insufficient stimulation of CD4^+^ T cells (Figure 5E,F). Furthermore, the expression profiles of genes associated with Th1 (T‐bet and IFN‐γ) exhibited reduction, while those indicative of Th2 (Gata3, IL‐4, IL‐13, and IL‐10) displayed elevation within the Th+5%Mn‐SiHANW group (Figure 5G). Flow cytometry data accentuated an augmented population of Gata3^+^ cells, coupled with a decline in T‐bet^+^ cells within the Th+5%Mn‐SiHANW group (Figure 5H–K).

These in vitro findings mirror the observations in the murine mandibular bone healing model, collectively endorsing that the presence of Mn fosters a milieu wherein naïve T helper cells undergo Th2 polarization while concurrently curbing Th1 polarization. This dynamic shift is paralleled by corresponding alterations in cytokine production, inducing an anti‐inflammatory microenvironment with elevated IL‐4 and decreased IFN‐γ. IL‐4 and IFN‐γ are the main cytokines that distinguish Th1 and Th2 cells, and control the balance of Th1 and Th2 cells. IL‐4 promotes Th2 cell development from naive helper T cells. Th2 cells create more IL‐4 as a result of being activated by IL‐4, creating a positive feedback loop.^[^ ^41^ ^]^ IFN‐γ is mostly secreted by Th1 cells, while on the flip side, it induces naive T helper cells to develop into Th1 cells. From an interaction standpoint, IL‐4 inhibits the generation of Th1 cells and IFN‐γ while IFN‐γ inhibits the development of Th2 cells. IFN‐γ and IL‐4 have critical roles in osteogenesis in addition to their immunoregulatory roles. It has been proven that IL‐4 stimulates osteoblast differentiation and bone regeneration.^[^ ^42^ ^]^ IFN‐γ plays a dual role in osteogenesis in a dose‐dependent manner.^[^ ^43^ ^]^

To corroborate the osteogenic effects of IL‐4 and IFN‐γ generated by Mn‐induced T helper cells, alkaline phosphatase (ALP) activity and osteogenic gene expressions of BMSC incubated in conditioned medium supplemented with differentiation cytokines and antibodies were analyzed (Figure 5L–N). Anti‐IL‐4 antibodies were administered to the Th+5%Mn‐SiHANW group, neutralizing the IL‐4 concentration. Consequently, the osteogenic effects were diminished, as evidenced by diminished ALP activity and osteogenic gene expression. Conversely, the addition of IL‐4 recombinant protein to the Th+SiHANW group augmented BMSC osteogenesis. The preceding results imply that IL‐4 is an efficient osteogenic factor produced by T helper cells stimulated with Mn‐HANW. IFN‐γ recombinant protein was additionally administered to the Th+5%Mn‐SiHANW group to mitigate the effect of Th1 function impairment. Moreover, ALP activity and osteogenic gene expression were significantly down‐regulated. These results indicated that IL‐4 and IFN‐γ are efficacious factors of Mn‐SiHANW–stimulated T helper cells and that IL‐4 induces osteogenesis in BMSC while IFN‐γ inhibits it.

### 2.5 Mn‐SiHANWs Increase Th2 and Inhibit Th1 Polarization under the Manipulation of MnSOD

The tunability of T helper cells can be effectively harnessed through various factors encompassing geography, parameters, scaffold structure, surface properties, chemical components, and stiffness of implanted biomaterials.^[^ ^35^, ^39^, ^44^ ^]^ Multiple sophisticated artificial antigen‐presenting cells (aAPC) were created by arranging receptor‐targeting cytokines and nanoparticles in intricate patterns.^[^ ^45^ ^]^ These types of aAPCs enable robust T cell activation. However, few of them are applied to bone tissue regeneration. Despite the fact that receptor‐targeting cytokines can support the adaptive immunomodulatory function of implanted biomaterials, challenges pertaining to stability, cost‐effectiveness, ease of manufacture, and controlled release persist, necessitating further refinement.^[^ ^45a^ ^]^ Against this backdrop, the biomaterial used in this study offers an alternate approach to the stated issues and is capable of modulating a certain subset of T cells to some extent during bone healing due to its controlled release of Mn, stable properties, bone‐mimicking crystal morphology, cost‐effectiveness, and ease of manufacture.

**Figure 6:**
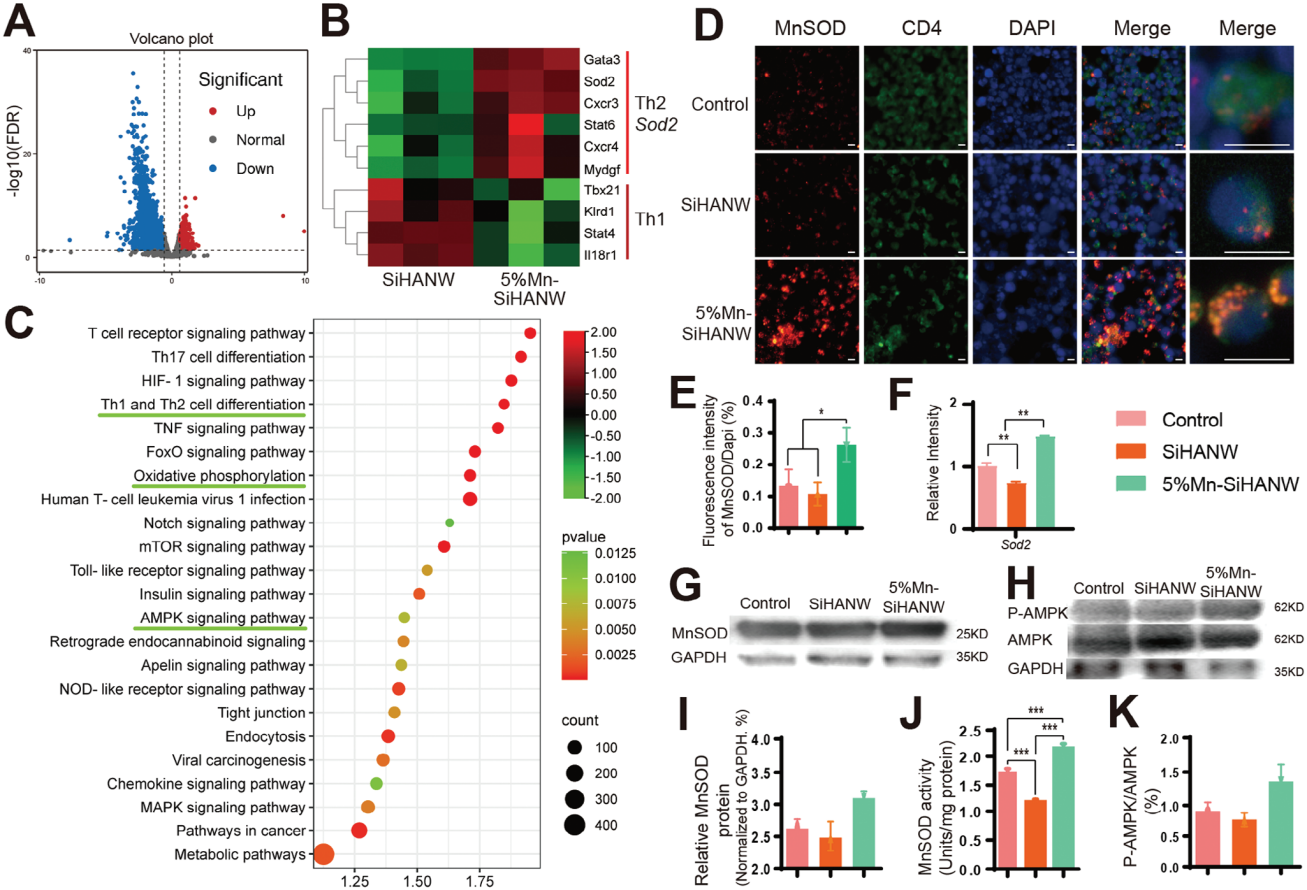
Manganese superoxide dismutase (MnSOD) role in Mn‐SiHANW–induced Th1/Th2 polarization. A) Volcano plot illustrating the differentially expressed genes between T cells treated with SiHANW or 5% Mn‐SiHANW in vitro. B) Hierarchical clustering of RNA‐sequence data indicating expression levels of Sod2 (MnSOD) and genes promoting Th1 or Th2 differentiation. Expression levels are indicated by color differences, as shown in the bottom bar. C) Pathway enrichment by KEGG analysis of differential genes. D) Immunofluorescence of MnSOD, CD4, and DAPI in CD4^+^ T cells treated with Control, SiHANW, and 5% Mn‐SiHANW. MnSOD: red; CD4: green; DAPI: blue. Scale bar: 20 µm. E) Fluorescence intensity of MnSOD/DAPI. F) Gene expression pattern of MnSOD. G) Western blot and H) protein level analysis of MnSOD. I) MnSOD activity of CD4^+^ T cells stimulated by PBS (Control), SiHANWs, or 5% Mn‐SiHANWs. J) Western blotting and K) protein levels of phosphorylated (P)‐AMPK and AMPK. *P < 0.05, **P < 0.01, ***P < 0.001.

In the following paragraphs, we will explore the mechanism underlying the effect of Mn‐SiHANWs on Th1/Th2 polarization. First, T helper cells were co‐cultured in vitro with PBS (Control), SiHANWs, and 5% Mn‐SiHANWs. Carboxy‐fluorescein‐succinimidyl‐ester analysis indicated that T helper cells proliferated actively in response to SiHANWs and 5% Mn‐SiHANWs between days 3 and 5 (Figure S4, Supporting Information). Subsequently, the SiHANW and 5% Mn‐SiHANW groups were subjected to RNA sequencing (**Figure** **6**A–C). A constellation of 1287 genes was up‐regulated, whereas 2652 genes were down‐regulated in the 5% Mn‐SiHANW group (Figure 6A). Among these disparate genes, superoxide dismutase 2 (Sod2*)*, or specifically MnSOD, along with genes related to Th2 cells, surfaced with heightened expression, while those associated with Th1 cells were down‐regulated in the 5% Mn‐SiHANW group (Figure 6B). This proposition is substantiated by RT‐PCR data showcasing a surge in MnSOD expression in the 5% Mn‐SiHANW group (Figure 6F). Intriguingly, CD4^+^ T cells treated with 5% Mn‐SiHANW exhibited robust cytoplasmic fluorescent signals indicative of MnSOD protein localization at a cytoplasmic pole (Figure 6D,E). Further corroborating this effect, western blot analysis revealed elevated MnSOD protein levels in the 5% Mn‐SiHANW group, in contrast to the SiHANW group, which displayed comparably nominal levels (Figure 6G,I). Notably, MnSOD is a critical enzyme in OXPHOS for maintaining redox status and energy metabolism. The MnSOD enzyme activity was enhanced prominently in the 5% Mn‐SiHANW group (Figure 6J). This finding indicated that the Mn‐existing milieu can activate the MnSOD enzyme, which may sequentially regulate the Th1/Th2 balance.

Under inflammatory conditions, MnSOD expression varies across diverse tissues.^[^ ^20a^ ^]^ Notably, it exerts divergent effects on multiple immune cells. For instance, escalated MnSOD levels coincide with the considerable suppression of pro‐inflammatory cytokines such as IL1b, IL‐6, and IL‐8.^[^ ^46^ ^]^ Further manifestations encompass heightened neutrophil antithrombotic functionality,^[^ ^47^ ^]^ attenuated CD8^+^ T cell response,^[^ ^48^ ^]^ augmented CD68^+^ macrophage recruitment within lung adenocarcinoma,^[^ ^49^ ^]^ and a proclivity toward M2 macrophage polarization in breast cancer.^[^ ^50^ ^]^ Our research indicates that MnSOD promotes the polarization of Th2 cells while inhibiting the polarization of Th1 cells, thereby completing the framework of MnSOD‐mediated immunology. Further research should elaborate on how mitochondrion behaves and OXPHOS changes under the Mn‐induced hyperfunction of MnSOD.

In the oral and maxillofacial environment, Mn‐doping biomaterials may provide a promising strategy for bone regeneration in cases of pathological disorders like periodontitis. These conditions are characterized by excessive pro‐inflammatory responses and a breakdown of the inflammatory balance.

### 2.6 Mn‐SiHANW Modulates the Th1/Th2 Balance by Targeting AMPK

To gain insight into how Mn‐SiHANW modulates the Th1/Th2 balance, a KEGG pathway enrichment analysis was performed. The results suggested that differential genes were enriched in Th1 and Th2 cell differentiation, OXPHOS, and the adenosine 5′‐monophosphate–activated protein kinase (AMPK) pathway (Figure 6C and Table S6, Supporting Information). Based on these results, it seems that Mn‐SiHANW may regulate the Th1/Th2 balance via AMPK‐related and OXPHOS‐involved energy metabolism.

Exploration into the underlying mechanics of T cell polarization, particularly concerning the modulation of T helper cells infiltrating local inflammatory microenvironments, has revealed the pivotal regulatory role of energy metabolism.^[^ ^51^ ^]^ AMPK is a pivotal orchestrator of energy metabolism integral to processes of anti‐inflammation and tissue regeneration.^[^ ^52^ ^]^ Within the energy metabolism continuum, AMPK signaling inhibits glycolysis, glutaminolysis, and fatty acid synthesis while supporting fatty acid oxidation and OXPHOS, thereby bolstering ATP production.^[^ ^53^ ^]^ Anomalies in OXPHOS were implicated in hampered Th2 responses in allergic asthma models.^[^ ^16^ ^]^ Conversely, glycolysis was implicated in fostering Th1‐driven IFN‐γ production within the inflamed area.^[^ ^17^ ^]^ Notably, fatty acid oxidation and ATP accumulation were associated with enhanced Th2 cell responses.^[^ ^18^ ^]^ In certain instances, AMPK activation has been reported to reduce the severity of dextran sodium sulfate‐induced colitis in mice via diminishing Th1 polarization,^[^ ^54^ ^]^ and also alleviating autoimmune encephalomyelitis by attenuating Th1‐favored IFN‐γ and TNF production while concurrently augmenting Th2‐primed IL‐4 and IL‐10.^[^ ^55^ ^]^ Furthermore, AMPK has proven pivotal in orchestrating the transition of M1 to M2 macrophages during muscle healing processes,^[^ ^56^ ^]^ further underscoring its influential role in shaping immunomodulated profiles conducive to Th2 amplification and Th1 attenuation.

**Figure 7:**
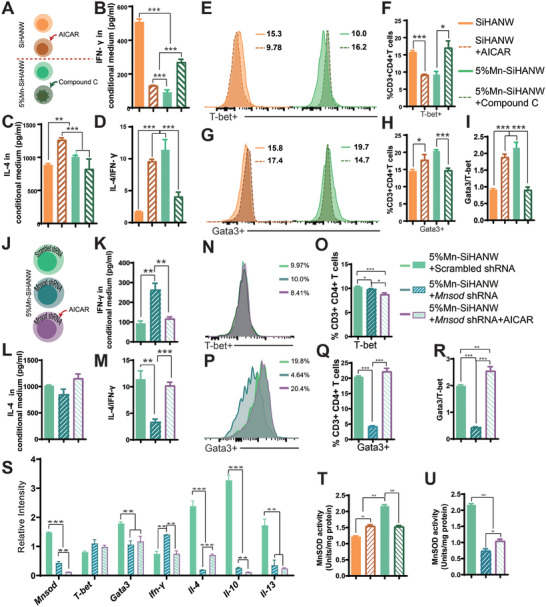
The MnSOD/adenosine 5′‐monophosphate‐activated protein kinase (AMPK) axis modulates Th1/Th2 balance in the Mn‐SiHANW–induced milieu. A) Experimental groups (SiHANWs, SiHANW+AMPK agonist 5‐aminoimidazole‐4‐carboxamide ribonucleoside [AICAR], 5% Mn‐SiHANW, and 5% Mn‐SiHANW+Compound C) for assessing Th1/Th2 balance upon stimulation with the AMPK activator or inhibitor. B) Protein level assessments concerning IL‐4, C) IFN‐γ, and D) IL‐4/IFN‐γ ratios in the conditioned medium. E,F) Transcriptional factor analysis involving G,H) Gata3, T‐bet, and I) Gata3/T‐bet in CD4^+^ T cells in response to four different stimuli. J) Experimental groups (5% Mn‐SiHANW+scrambled shRNA, 5% Mn‐SiHANW+MnSOD shRNA, and 5% Mn‐SiHANW+MnSOD shRNA+AICAR) elucidating MnSOD role and its connection to the AMPK signaling pathway in Th1/Th2 balance. K) Protein level assessments concerning IL‐4, L) IFN‐γ, and M) IL‐4/IFN‐γ ratios in the conditioned medium. N,O) Transcriptional factor analysis involving Gata3, P,Q) T‐bet, and R) Gata3/T‐bet in CD4^+^ T cells under three different stimuli. S) Relative intensity of MnSOD, T‐bet, Gata3, IFN‐γ, IL‐4, IL‐10, and IL‐13 under different stimuli. T) MnSOD activities of CD4^+^ T cells treated with an AMPK activator or inhibitor. U) MnSOD activities in 5% Mn‐SiHANW+scrambled shRNA, 5% Mn‐SiHANW+MnSOD shRNA, and 5% Mn‐SiHANW+MnSOD shRNA+AICAR groups. *P < 0.05, **P < 0.01, ***P < 0.001.

Thus, we sought to verify the specific role of Mn‐SiHANW–induced AMPK activation in the Th1/Th2 balance. The ratio of phosphorylated‐AMPK (p‐AMPK)/AMPK was detected in the presence of PBS, SiHANW, and 5%Mn‐SiHANW. The ratio of p‐AMPK/AMPK rose in the 5% Mn‐SiHANW group, whereas it fell in the SiHANW group (Figure 6H,K). To determine whether AMPK activation could restore the Th1/Th2 balance in the SiHANW group, 5‐aminoimidazole‐4‐carboxamide ribonucleoside (AICAR) was added to the SiHANW group (SiHANW+AICAR). While Compound C was applied to inhibit the AMPK pathway in 5%Mn‐SiHANW–treated CD4^+^ T cells (5%Mn‐SiHANW+Compound C) (**Figure** **7**A).

To analyze the functional change, the conditioned medium was assembled for IL‐4 and IFN‐γ quantification. The 5%Mn‐SiHANW and SiHANW+AICAR groups contained more IL‐4 and less IFN‐γ than those in the SiHANW group, indicating that the introduction of Compound C significantly increased IFN‐γ while inhibiting IL‐4 secretion (Figure 7B–D). T‐bet and Gata3 were quantified using flow cytometry. Notably, the 5%Mn‐SiHANW+Compound C group displayed augmented proportions of T‐bet+ cells, whereas the SiHANW+AICAR cohort evinced a decline in T‐bet^+^ cell proportions (Figure 7E,F). In contrast to T‐bet^+^ cells, histograms and statistical analysis revealed an inverted trend in Gata3^+^ cells, with AICAR treatment augmenting Gata3^+^ cell proportions in the SiHANW group, while Compound C addition to the 5%Mn‐SiHANW group curtailed Gata3^+^ cell proportions (Figure 7G,I).

In light of the cumulative functional and transcriptional factor analyses, it becomes apparent that AMPK signaling potently tips the Th1/Th2 balance in favor of Th2 polarization rather than Th1 polarization. Nevertheless, this work did not incorporate an investigation into the response of energy metabolism to AMPK activation under conditions when Mn is present. Future studies will aim to provide a more comprehensive understanding of the metabolic alterations associated with OXPHOS, glycolysis, and fatty acid metabolism.

### 2.7 Mn‐SiHANW Modulates Th1/Th2 Balance via the MnSOD/AMPK Pathway

MnSOD serves as the upstream regulator of AMPK in multiple circumstances. MnSOD sustains AMPK activation in aggressive breast cancer^[^ ^57^ ^]^ and colorectal cancer.^[^ ^58^ ^]^ Remarkably, compounds such as quercetin promote AMPK protein phosphorylation through the up‐regulation of MnSOD expression, augmenting osteogenic differentiation, and the antioxidant response within BMSC.^[^ ^59^ ^]^ In the Control, SiHANW, and 5%Mn‐SiHANW groups, the relative protein levels of MnSOD were found to be proportional to the phosphorylated (P)‐AMPK levels (Figure 6I,K). These clues prompted us to assume a regulatory relationship between MnSOD and the AMPK pathway in 5%Mn‐SiHANW–induced Th1 and Th2 polarization.

With the aim of substantiating this hypothesis, MnSOD shRNA was employed to impair MnSOD expression within CD4^+^ T cells (MnSOD shRNA group), while scrambled shRNA served as a control (scrambled shRNA group). Subsequently, AICAR was introduced to the MnSOD shRNA group to ascertain whether AMPK activation could rectify the dwindling Th2‐to‐Th1 population ratio (Figure 7J). Following a 3 days incubation period within 5%Mn‐SiHANWs, the three groups underwent functional assessments, encompassing quantification of IL‐4 and IFN‐γ protein levels. Impressively, the 5%Mn‐SiHANW+MnSOD shRNA group exhibited minimal IL‐4 secretion juxtaposed against heightened IFN‐γ levels. Notably, AMPK activation within the 5%Mn‐SiHANW+MnSOD shRNA group abrogated the altered IL‐4/IFN‐γ ratio, restoring it to baseline levels (Figure 7K–M). Concomitantly, the proportion of Gata3^+^ cells was attenuated within the 5%Mn‐SiHANW+MnSOD shRNA group, yet reinvigorated upon supplementary AICAR stimulation (Figure 7P,Q). Curiously, T‐bet^+^ cell proportions remained unaltered in response to similar stimuli (Figure 7N,O). Comparatively, the relative mRNA expression pattern in the 5%Mn‐SiHANW+MnSOD shRNA group revealed a decrement in Sod2, Gata3, IL‐4, IL‐10, and IL‐13 expressions, contrasted by heightened T‐bet and IFN‐γ levels, particularly when AICAR was introduced (Figure 7S). After AICAR was additionally added, Gata3 and IL‐4 increased, while T‐bet and IFN‐γ decreased (Figure 7S). Based on the preceding results, the AMPK signaling pathway acted downstream of MnSOD in the Th1/Th2 balance in the Mn‐existing milieu.

This research unveils intriguing parallels with previously documented mutual interactions between MnSOD and AMPK, reinforcing the presence of a similar pathway within our investigative purview. Remarkably, AMPK activation in the SiHANW group was also associated with heightened MnSOD activity (Figure 7T). Conversely, in the 5% Mn‐SiHANW group, AMPK inhibition elicited a decline in MnSOD activity (Figure 7T). Noteworthy observations unfolded upon MnSOD shRNA infection, where MnSOD activity witnessed a notable decline, subsequently exhibiting a measured resurgence upon exposure to AICAR (Figure 7U). Collectively, these outcomes establish AMPK as a downstream influencer of MnSOD, as underscored by its intricate and inverse modulation of MnSOD protein activity.

## 3 Conclusion

The intricate orchestration of T cell polarization has emerged as a focal point of interest, finding application across diverse therapeutic domains. Employing ingeniously tailored biomaterials to finely regulate the equilibrium between pro‐inflammatory and anti‐inflammatory T cell subsets stands as a promising avenue for driving comprehensive bone regeneration. Within the ambit of this investigation, we harnessed the potency of Mn ion incorporation into Si‐hydroxyapatite nanowires, propelling osteogenic outcomes through two pivotal mechanisms: 1) Mn, administered at an optimal dosage, effectively stimulates BMSC to engage in proficient osteogenic differentiation and robust mineralization; 2) The strategic inclusion of Mn imparts an anti‐inflammatory and regenerative milieu by adroitly modulating the Th1/Th2 cell equilibrium and the attendant cytokine repertoire, thereby markedly augmenting the osteogenic trajectory. Throughout this process, the MnSOD/AMPK pathway emerges as a linchpin, orchestrating the Mn‐induced immunomodulatory dynamics. This exposition underscores the fact that Mn‐doping amplifies the osteogenic efficacy of biomaterials, assuming the role of a precision immunoregulator in the intricate landscape of bone defect healing. It follows that the Mn‐SiHANW framework holds auspicious prospects for clinical translation, accentuated by attributes including facile and cost‐effective manufacturing, structural stability, and commendable biocompatibility.

## 4 Experimental Section

### Synthesis and Characterization of Si‐Hydroxyapatite Nanowires with or without Mn‐Doping

The elementary method of synthesizing Si‐hydroxyapatite nanowires with or without Mn‐doping was detailed in the work by Lin et al.^[^ ^60^ ^]^ Briefly, Ca(NO_3_)_2_·4H_2_O, MnCl_2_, CaCl_2_, and Na_2_SiO_3_·9H_2_O were solubilized in distilled water to initiate the formation of precursors through a hydrothermal route. These precursors were devised with distinct Mn/(Ca+Mn) mole ratios, specifically 0, 0.05, and 0.1, corresponding to the fabrication of SiHANWs, 5% Mn‐SiHANWs, and 10% Mn‐SiHANWs. The ensuing process encompassed thorough rinsing with distilled water and anhydrous ethanol, succeeded by drying to eliminate any residual moisture. The prepared precursors were subsequently amalgamated with an aqueous solution of Na_3_PO_4_, subjected to a 120 °C heat treatment for 24 h, and then processed through sequential steps of washing, filtration, and 180 °C drying for an additional 24 h.

The architectural attributes and elemental distribution of Ca, Mn, and Si were scrutinized employing SEM (ZEISS GeminiSEM 300, Germany) and TEM (FEI Talos F200S, USA). Thorough physicochemical evaluations were conducted. Infrared spectra analysis was conducted on small pellets containing 1 mg of the sample, encompassing a spectral range of 500 to 4000 cm^−1^, employing FTIR (Thermo Scientific Nicolet iS5, USA). The crystalline structures were meticulously examined using XRD (Bruker D8 Advance, Germany) with a scanning velocity of 0.02°, an operational voltage of 40 kV, and a 2*θ* range spanning from 10° to 80°. The quantification of Mn and Ca ions leached over specified durations (days 1, 3, 7, 14, 21, and 28) was executed via ICP‐OES (Agilent 730 [OES], USA). In a concise outline, 5 mg of the sample was suitably diluted with 10 mL of PBS solution (pH 7.4) and incubated at 37 °C. Subsequent calculations encompassed the determination of Mn/(Ca+Mn) mole ratios, accompanied by recording pH values at predefined intervals.

### Proliferation Activity and Morphology Analysis of BMSC

BMSCs were extracted from the long bones of male C57BL/6 mice (3 weeks old). The proliferation of BMSC was evaluated through the deployment of the CCK‐8 assay (Dojindo, Japan). BMSCs were seeded at a density of 3 × 10^3^ cells per well in 96‐well flat‐bottomed cell culture plates and exposed to varying concentrations (0.1, 1, 10, and 100 µg mL^−1^) of SiHANWs, 5% Mn‐SiHANWs, and 10% Mn‐SiHANWs suspending solutions. On days 1, 3, and 7, 10 µL of CCK‐8 solution were introduced to each well and incubated at 37 °C for an hour. Subsequently, absorbance readings were recorded at 450 nm to quantify the proliferation activity of BMSC. The impact of distinct concentrations of SiHANWs, 5% Mn‐SiHANWs, and 10% Mn‐SiHANWs on the morphology of BMSC was evaluated through dual‐staining utilizing phalloidin and 4′6‐diamidino‐2‐phenylindole (DAPI, 1:200, Thermo Fisher Scientific, Warsaw, Poland). This visualization was facilitated by confocal laser scanning microscopy (Leica, Germany).

### Osteogenic Differentiation Analysis

The assessment of osteogenic differentiation was conducted by cultivating BMSC in distinct conditioned mediums. The expression levels of Runx2, osteonectin, Opn, and Collagen I in BMSC were scrutinized through RT‐PCR on the 7^th^ day of culture. Total RNA was extracted from CD4^+^ T cells utilizing TRIzol (Invitrogen, Japan), followed by reverse transcription using PrimeScript reverse transcriptase (Takara, Japan). RT‐PCR was executed on the LC96 platform (Roche) with the application of TB Green Premix Ex Taq (Tli RNaseH Plus, Takara, Japan). The specific primers employed for real‐time PCR are enlisted in Table S7, Supporting Information. The quantification of target gene expressions was achieved using the 2^−ΔΔCt^ method, employing GAPDH as the internal reference gene.

Qualitative assessment of ALP activity was conducted through the BCIP/NBT alkaline phosphatase color development kit, whereas the alkaline phosphatase assay kit (Beyotime, China) enabled quantitative measurement. Alizarin Red S staining kit (Beyotime, China) was used to observe the mineralization of BMSC. The amount of calcium deposits was measured after adding 100 nm cetylpyridinium chloride. The absorbance was measured at 562 nm.

### CD4^+^ T Cell‐Depleted Murine Model and Mandibular Defect Surgery

Male C57BL/6 mice (8 weeks old) were purchased from Vital River Laboratory Animal Technology (Beijing, China). The animals were kept in a 12/12 hours dark/light cycle at 25 °C. C57BL/6 (male, 8 weeks) were housed in accredited animal facilities in specific pathogen‐free conditions at Shanghai Ninth People's Hospital (Shanghai, China). All animal experiments were conducted in strict compliance with the guidelines of the Animals Committee of Shanghai Ninth People's Hospital (SH9H‐2023‐A802‐1).

The mice were randomly divided into two groups: the CD4^+^ T cell‐depleted group, subjected to intravenous administration of anti‐CD4 antibodies, and the control group, treated with saline. After a 3 weeks interval, CD4^+^ T cells were collected from blood cells and quantified using flow cytometry to verify whether the model was established successfully.^[^ ^61^ ^]^

Murine mandibular bone defects were generated according to the authors' previous article.^[^ ^29^ ^]^ Briefly, mice were anesthetized through the inhalation of 2% isoflurane. Subsequently, the masseter muscles were meticulously dissected between the superior and inferior branches of the facial nerve following a 5 mm mandibular skin incision, followed by an incision of the periosteum. Employing a dental bur, full‐thickness penetrating defects with a diameter of 2.3 mm were meticulously fashioned, with continuous saline irrigation for cooling. Subsequent to cleansing of the surgical site with saline, the surgical defect was loaded with 5 µL of gelatin methacryloyl (Gelma, EFL‐GM, Yongqinquan Intelligent Equipment Co., Ltd., China)‐loaded solutions encompassing PBS, SiHANW, 5% Mn‐SiHANW, and 10% Mn‐SiHANW (referred to as Gelma, Gelma+SiHANW, Gelma+5%Mn‐SiHANW, and Gelma+10%Mn‐SiHANW). The surgical site was subsequently sealed utilizing a 5‐0 suture. Euthanasia of timed mice was executed through CO_2_ administration, followed by a thoracotomy procedure to ensure complete cessation of vital functions.

### Micro‐CT and Histological Examination

Eight weeks post‐implantation, C57BL/6 mice were humanely euthanized, and their mandibles were excised and subjected to fixation in 4% paraformaldehyde. Subsequent Micro‐CT scanning of the mandibles was conducted utilizing the SkyScan 1076 system (Belgium) at a spatial resolution of 24 µm. For the purpose of 3D reconstruction, gray scale settings were adjusted within the range of 120 to 255. Parameters include BV/TV, and BMD was computed. For histological evaluation, specimens were sectioned to a thickness of 4 µm, followed by processing for standard hematoxylin and eosin (H&E) staining and Masson staining. Immunohistochemistry analysis was performed after employing heat‐mediated antigen retrieval using sodium citrate buffer (pH = 6, ab208572, abcam, UK) for a duration of 20 min. The application of primary antibodies targeting OCN protein (1:150, 23418‐1‐AP, proteintech, UK) ensued, and after an overnight incubation at 4 °C, sections were visualized using an HRP‐conjugated compact polymer system, with DAB serving as the chromogen. Visual documentation of all stained paraffin sections was achieved via Olympus microscopy.

### CD4^+^ T Cell Subset's Functional Analysis

Functional analysis of CD4^+^ T cell subsets was conducted utilizing flow cytometry analysis and ELISA techniques. Mice were euthanized at postoperative intervals of 1, 4, or 7 days following the mandibular defect surgery described earlier. Blood and mandibular bone defect site samples were collected. Bone tissue was gently triturated, and the resultant cellular suspension was filtered through a 70 µm filter (BD Falcon). Red blood cells were lysed using an ammonium‐chloride–potassium buffer. The inclusion of a Live/Dead kit (BV510, Zombie Aqua fixable viability kit, BioLegend), CD45 (Alexa Flour 700, BioLegend, USA), CD3 (APC‐Cy7, BD Biosciences, USA), and CD4 (PerCP‐Cy5.5, BioLegend) was executed as per manufacturers’ protocols. Following fixation and permeabilization using a flow cytometry permeabilization/wash buffer (BD Biosciences), Gata3 (APC, BioLegend), and T‐bet (BV421, BioLegend) antibodies were introduced. Stained cells were subsequently analyzed using a BD LSRFortessa flow cytometer, and data interpretation was accomplished utilizing Flowjo software (BD Biosciences). The gating strategy is elucidated in Figure S4A, Supporting Information.

In the context of ELISA, the supernatant extracted from the ground mandibular defect area was subjected to examination. Concentrations of IL‐4 and IFN‐γ were quantified employing ELISA kits (R&D Systems) as per the manufacturer's instructions.

### CD4^+^ T Cell Culture and Assessment

A single‐cell suspension derived from the spleen of male C57BL/6 mice (8 weeks old) was prepared for the isolation of CD4^+^ T cells employing MojoSort mouse CD4 nanobeads (BioLegend, USA). The isolated T cells were resuspended in RPMI 1640 medium supplemented with 10% fetal bovine serum and 1% penicillin–streptomycin (HyClone, Thermo Scientific). These cells were cultured in humidified air comprising 5% CO_2_ at 37 °C. Subsequently, the cells were stimulated with anti‐CD3 antibodies (5 µg mL^−1^, BioLegend) and anti‐CD28 antibodies (2 µg mL^−1^, BioLegend), followed by incubation with PBS (Control), SiHANWs, or 5% Mn‐SiHANWs at a concentration of 1 µg mL^−1^ within RPMI 1640 media, and incubated at 37 °C for a duration of 72 h. After the 72 h incubation period, the conditioned media was collected from the supernatant of the CD4^+^ T cells by passing through PES filters (Merck Millipore).

Subsequent quantification of IL‐4 and IFN‐γ concentrations was accomplished using ELISA techniques. Moreover, CD4^+^ T cells were retrieved and subjected to flow cytometry analysis. Prior to the inclusion of CD3 (APC‐Cy7, BD Biosciences, USA), CD4 (PerCP‐Cy5.5, BioLegend), Gata3 (APC, BioLegend), and T‐bet (BV421, BioLegend) antibodies, cells were treated with a Live/Dead kit (BV510, Zombie Aqua fixable viability kit, BioLegend). The gating strategy is delineated in Figure S5B, Supporting Information. The specific primers for RT‐PCR targeting the genes under examination are detailed in Table S7, Supporting Information. The expression of target genes was calculated.

### MnSOD Expression and Activity

MnSOD gene expression was analyzed by RT‐PCR using a forward MnSOD primer (5′‐GTTGTGTCCTTTTTTGTCACC‐3′) and a reverse MnSOD primer (5′‐TTCCTGTCTTTTCCTCCCC‐3′).

Western blotting was employed to analyze the protein levels of MnSOD. In brief, CD4^+^ T cells stimulated by PBS (Control), SiHANWs, or 5%Mn‐SiHANWs were harvested, cold PBS‐washed, and subsequently lysed with ice‐chilled RIPA buffer containing protease and phosphatase inhibitors for a duration of 10 min. The resulting lysates were then subjected to centrifugation at 120 000 rpm for 5 min. Protein concentrations were determined utilizing the BCA protein assay kit (Beyotime). Subsequently, the protein bands were incubated overnight at 4 °C with MnSOD mouse monoclonal antibody (1:1000, Cell Signaling Technology, USA) and GAPDH (1:1000, Proteintech). Horseradish‐peroxidase–conjugated secondary antibodies (Proteintech) were applied to the samples for 1 h at room temperature. Following washing with tris buffered saline tween, the hybridized bands were visualized through the utilization of the highly sensitive ECL (Thermo Fisher Scientific, USA).

Immunofluorescence staining was implemented to visualize the MnSOD protein. CD4^+^ T cells were labeled with FITC anti‐mouse CD4 (BioLegend) antibody for 30 min and subjected to three cold PBS washes. These cells were then fixed on slides with 4% paraformaldehyde and permeabilized with 0.2% Triton X‐100 in PBS for a duration of 15 min. After a 1 h blocking step with 1% BSA at room temperature, the samples were incubated overnight at 4 °C with MnSOD mouse monoclonal antibody (1:200, Cell Signaling Technology, USA). Subsequent incubation with Cy3‐conjugated goat anti‐mouse IgG (1:200, Proteintech) was performed for 1 h at room temperature. Nuclei were stained with DAPI (BioLegend).

The activity of MnSOD was gauged utilizing the Cu/Zn‐SOD and MnSOD assay kit with WST‐8 (Beyotime). In a nutshell, CD4^+^ T cells were washed and homogenized with cold PBS. Subsequent determination of protein concentration was conducted using the BCA protein assay kit (Thermo Scientific). Samples were subjected to incubation with Cu/Zn‐SOD inhibitors at 37 °C, followed by incubation with a working solution. Absorbance measurements were taken at 562 nm. One unit of SOD was defined as the amount of MnSOD required to neutralize 50% of the superoxide radical.

### RNA Sequencing and Lentivirus Knockdown of MnSOD

Total RNA was isolated from the mandibular defect area subjected to distinct GelMa‐loaded SiHANWs and 5% Mn‐SiHANWs, as well as CD4^+^ T cells cultured with SiHANWs or 5% Mn‐SiHANWs. TRIzol reagent (Takara, Japan) was employed for this purpose. The sequencing platform utilized was the Illumina HiSeq X Ten platform, with differential expression analysis carried out using the DESeq (2012) R package. Genes displaying a significance threshold of *P* < 0.05 were classified as differentially expressed. Enrichment of pathways was performed through KEGG (http://www.kegg.com). The CellMarker database (http://biocc.hrbmu.edu.cn/CellMarker/ or http://bio‐bigdata.hrbmu.edu.cn/CellMarker/) was used for distinguishing different immune cell types in tissues.^[^ ^62^ ^]^

The MnSOD shRNA lentivirus was constructed by Genomeditech (Shanghai, China). Primary CD4^+^ T cells were infected with Mnsod shRNA lentivirus at 5 µg mL^−1^. The culture medium was replaced after 16 h of infection.

### AMPK Pathway Analysis and In Vitro Small Molecular Treatment

Western blotting was used to assess the protein levels of AMPK and p‐AMPK. Protein bands were exposed to AMPK mouse monoclonal antibody (1:1000, Cell Signaling Technology), P‐AMPK mouse monoclonal antibody (1:1000, Cell Signaling Technology), and GAPDH (1:1000, Proteintech) overnight at 4 °C. The subsequent steps had been previously outlined.

CD4^+^ T cells stimulated with SiHANWs or 5% Mn‐SiHANWs were subjected to incubation with AICAR (0.1 mm) or Compound C (1.25 µm) (Sellect, USA) for a duration of 3 days, followed by subsequent analyses.

### Statistical Analysis

For statistical analysis, SPSS 23.0 software was employed. Unpaired Student's *t*‐test was utilized for pairwise comparisons. A one‐way analysis of variance was conducted, followed by Tukey's multiple comparison test for comparisons involving more than two groups. Data are represented as the mean ± standard deviation (SD) from a minimum of three independent experiments. A significance level of *P* < 0.05 was considered statistically significant.

## Conflict of Interest

The authors declare no conflict of interest.

## Supporting information

Supporting Information

## Data Availability

The data that support the findings of this study are available from the corresponding author upon reasonable request.
