# Supplementary material for: Manganese Enhances the Osteogenic Effect of Silicon‐Hydroxyapatite Nanowires by Targeting T Lymphocyte Polarization
Source: Adv Sci (Weinh). 2023 Dec 1;11(4):2305890. doi: 10.1002/advs.202305890 (PMC10811488; doi:10.1002/advs.202305890)
Supplement: Supplementary file 1 — Supporting Information [file ADVS-11-2305890-s001.pdf]

ADVANCED SCIENCE
Open Access

Supporting Information

for *Adv. Sci.*, DOI 10.1002/advs.202305890

Manganese Enhances the Osteogenic Effect of Silicon-Hydroxyapatite Nanowires by Targeting T Lymphocyte Polarization

Ruomei Li, Zhiyu Zhu, Bolin Zhang, Ting Jiang, Cheng Zhu, Peng Mei, Yu Jin, Ruiqing Wang, Yixin Li, Weiming Guo, Chengxiao Liu, Lunguo Xia and Bing Fang**

Supporting Information

Manganese Enhances the Osteogenic Effect of Silicon-Hydroxyapatite Nanowires by Targeting T Lymphocytes Polarization

Ruomei Li, Zhiyu Zhu, Bolin Zhang, Ting Jiang, Cheng Zhu, Peng Mei, Yu Jin, Ruiqing Wang, Yixin Li, Weiming Guo, Lunguo Xia, and Bing Fang**

Table S1. The diameter and length of silicon-hydroxyapatite nanowire (SiHANW), 5% manganese-doped silicon-hydroxyapatite nanowire (Mn-SiHANW), and 10%Mn-SiHANW.

	Diameter (nm)				Length (nm)			
	Mean	SD	Min	Max	Mean	SD	Min	Max
SiHANW	14.35	2.92	9.12	21.44	128.22	36.67	50.33	212.52
5%Mn-SiHANW	19.34	3.95	11.21	29.47	212.6	85.72	52.57	449.44
10%Mn-SiHANW	13.98	3.75	6.37	23.48	131.61	43.44	67.14	204.16

SD, standard deviation; Min, Minimum; Max, maximum

Table S2. The mole ratios of Manganese (Mn)/(Mn+Calcium(Ca)) in SiHANW, 5%Mn-SiHANW, and 10%Mn-SiHANW.

	Mn/(Mn+Ca)				Mean	SD
5%Mn-SiHANW	0.068136	0.063579	0.066746	0.068444	0.066726	0.002224
10%Mn-SiANW	0.109576	0.091587	0.101928		0.10103	0.009028

SD, standard deviation

Table S3. The mole ratios of Mn/Mn+Ca for SiHANW, 5%Mn-SiHANW, and 10%Mn-SiHANW from ICP-OES/MS result.

	SiHANW	5%Mn-SiHANW	10%Mn-SiHANW
Day 1	0	0.005469146	0.016658081
Day 3	0	0.005300115	0.015261421
Day 7	0	0.008204745	0.015805165

Day 14	0	0.010584283	0.017844365
Day 21	0	0.013321593	0.025352023
Day 28	0	0.022449039	0.032686093

Table S4. Measurements of bone volume/total tissue volume (BV/TV) and bone mineral density (BMD) of Gelma Hydrogels (Gelma), Gelma+SiHANW, Gelma+5% Mn-SiHANW, and Gelma+10% Mn-SiHANW in mandibular bone healing.

	BV/TV			BMD		
	Mean	SD	*P*[a)]	Mean	SD	*P*[a)]
Gelma	25.26616	3.147114	<0.0001	0.691482	0.027224	0.0001
Gelma+SiHANW	34.1949	3.562202	0.0088	0.790488	0.026616	0.0002
Gelma+5%Mn-SiHANW	41.19612	4.337115	NA	1.041348	0.135941	NA
Gelma+10%Mn-SiHANW	27.04273	3.584622	0.0002	0.742233	0.065122	0.0007

SD, standard deviation; [a)], *P*-values of unpaired Student's t-test comparing each group to the Gelma+5%Mn-SiHANW group.

Table S5. Measurements of bone volume/total tissue volume (BV/TV) and bone mineral density (BMD) of Gelma Hydrogels (Gelma)+5% Mn-SiHANW+saline and Gelma+5% Mn-SiHANW+anti-CD4 in mandibular bone healing.

	BV/TV			BMD		
	Mean	SD	*P*	Mean	SD	*P*
Gelma+5%Mn-SiHANW+saline	37.90175	5.235767	0.0038	1.00251	0.157454	0.0014
Gelma+5%Mn-SiHANW+anti-CD4	21.4517	2.781758		0.551335	0.077672	

SD, standard deviation; *P*, *P*-values of unpaired Student's t-test.

Table S6. Kyoto Encyclopedia of Genes and Genomes (KEGG) analysis

	Count	%	*P*-Value	Fold Enrichment	FDR
T cell receptor signaling pathway	47	1.002774	1.39E-06	1.952408	1.21E-05
Th17 cell differentiation	47	1.002774	2.67E-06	1.915219	1.96E-05
HIF-1 signaling pathway	50	1.06678	2.50E-06	1.876614	1.95E-05
Th1 and Th2 cell differentiation	38	0.810753	7.32E-05	1.847612	3.37E-04
TNF signaling pathway	49	1.045445	8.39E-06	1.82309	5.19E-05
FoxO signaling pathway	53	1.130787	1.98E-05	1.731069	1.08E-04
Human T-cell leukemia virus 1 infection	100	2.133561	4.73E-09	1.711472	7.41E-08
Oxidative phosphorylation	54	1.152123	2.38E-05	1.711472	1.22E-04
Notch signaling pathway	24	0.512055	0.012668	1.629974	0.028085
mTOR signaling pathway	59	1.258801	7.94E-05	1.607912	3.59E-04
Toll-like receptor signaling pathway	36	0.768082	0.005409	1.540325	0.013522
Insulin signaling pathway	49	1.045445	0.001744	1.508312	0.005694
AMPK signaling pathway	43	0.917431	0.007758	1.448687	0.018604
Retrograde endocannabinoid signaling	50	1.06678	0.004165	1.4455	0.011653
Apelin signaling pathway	46	0.981438	0.006887	1.436637	0.016858

NOD-like receptor signaling pathway	72	1.536164	8.12E-04	1.426227	0.002936
Tight junction	55	1.173459	0.004758	1.409146	0.012821
Endocytosis	88	1.877534	6.06E-04	1.384279	0.002297
Viral carcinogenesis	73	1.557499	0.002829	1.363946	0.008522
Chemokine signaling pathway	60	1.280137	0.010856	1.337088	0.024529
MAPK signaling pathway	92	1.962876	0.003541	1.303439	0.010149
Pathways in cancer	161	3.435033	4.32E-04	1.268633	0.001663
Metabolic pathways	426	9.088969	0.00155	1.12583	0.005205

Table S7. Primers used for reverse transcription polymerase chain reaction (RT-PCR) primer sequencing.

Name	Sequence Forward (5’-3’)	Sequence Reverse (5’-3’)
Runx2	TACTTCGTCAGCATCCTATCAG	CAGCGTCAACACCATCATTC
Osteonectin	TGGGAGAATTTGAGGACGGTG	GAGTCGAAGGTCTTGTTGTCAT
OCN	AGGAGGGCAATAAGGTAGTG	TGTAGGCGGTCTTCAAGC
OPN	GACGATGATGACGATGATGATG	CGACTGTAGGGACGATTGG
Collagen I	CACCCTCAAGAGCCTGAGTC	GTTCGGGCTGATGTACCAGT
Mnsod	GTTGTGTCCTTTTTTGTCACC	TTCCTGTCTTTTCCTCCCC
T-bet	TCCCATTCCTGTCCTTCACCG	ATGCTGCCTTCTGCCTTTCCA
Gata3	CCTTTATTCCTCCGTGTCTGC	ATCTTTGCGGGATAGTTTAGC
Ifn-γ	GGTCCAGCGCCAAGCAT	GCTGGATTCCGGCAACAG
Il-4	GTTCGGGCTGATGTACCAGT	ACCTTGGAAGCCCTACAGACGAG
Il-10	CAGAGAAGCATGGCCCAGAAATC	TCTTCACCTGCTCCACTGCCTTG
Il-13	CGGCAGCATGGTATGGAGTG	ATTGCAATTGGAGATGTTGGTCAG
Gapdh	TCAACGGCACAGTCAAG	ACTCCACGACATACTCAGC

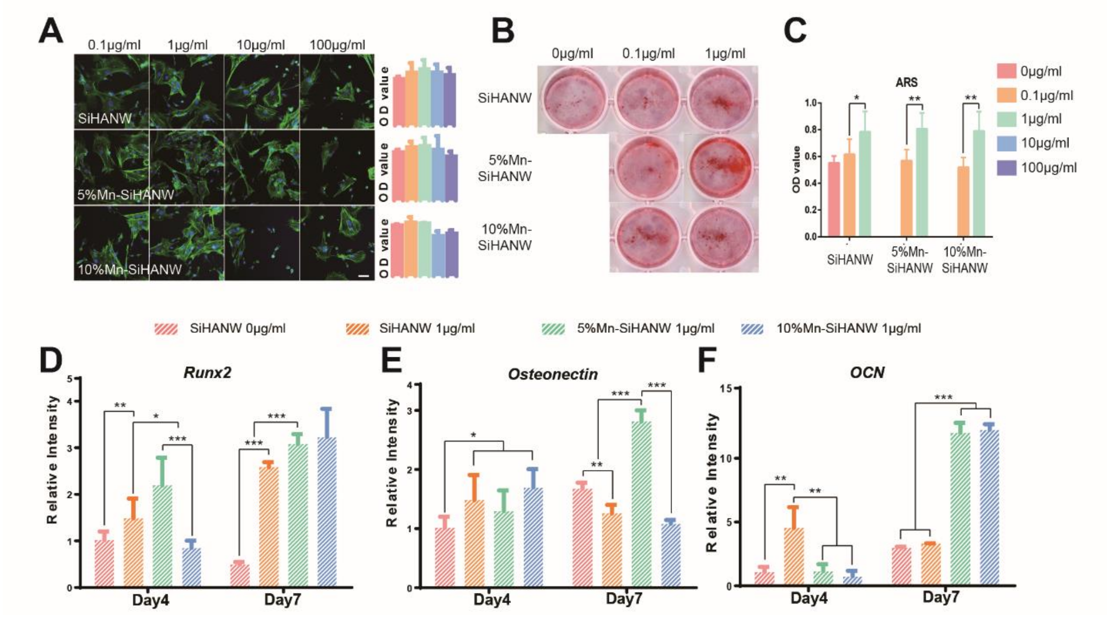

Figure S1. The biologic performance of SiHANW, 5% Mn-SiHANW, and 10%Mn-SiHANW. A) The morphologies and cell counting kit-8 (CCK-8) evaluation of bone marrow stromal cells (BMSC) co-cultured with SiHANWs, 5% Mn-SiHANWs, and 10%Mn-SiHANWs. Scale bar, 50 μm. Images (B) and quantifications (C) of safranin-O staining of BMSCs co-cultured with SiHANWs, 5% Mn-SiHANWs, and 10%Mn-SiHANWs at different concentrations. Runt-relate transcription factor 2 (*Runx2)* (D), *Osteonectin* (E), and Osteocalcin (*OCN)* (F) expressions of BMSC co-cultured with SiHANWs, 5% Mn-SiHANWs, and 10%Mn-SiHANWs. **P* < 0.05, ***P* < 0.01, ****P* < 0.001.

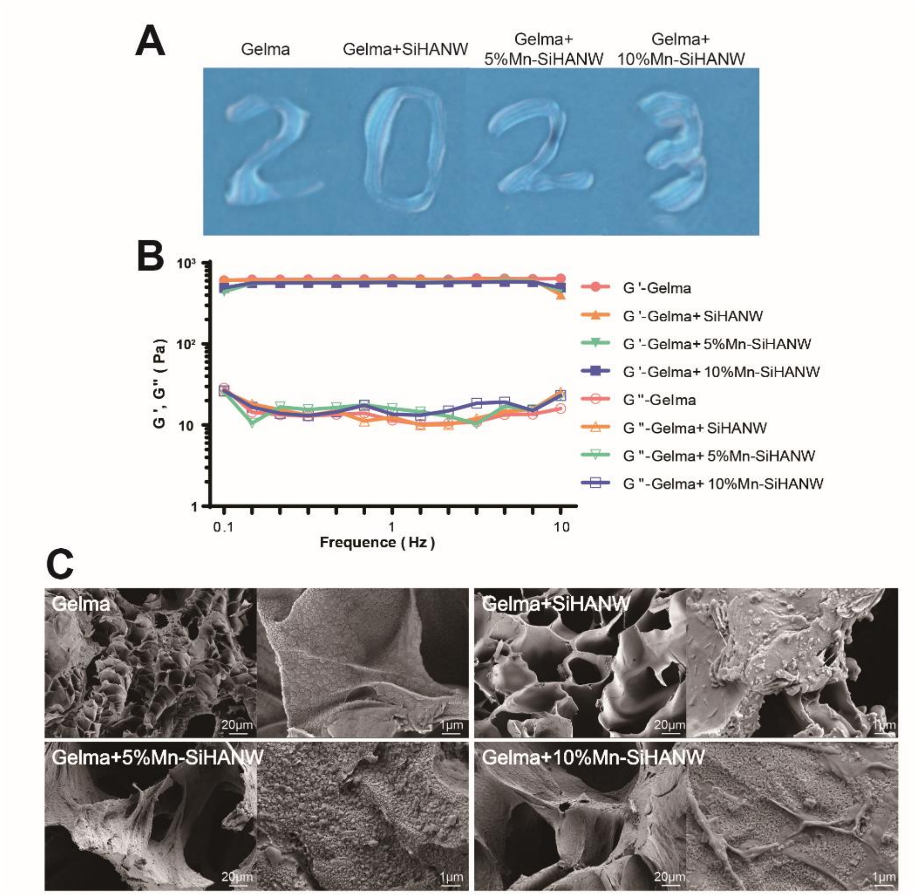

Figure S2. Characteristics of Gelma, Gelma+SiHANW, Gelma+5%Mn-SiHANW, and Gelma+10%Mn-SiHANW. The injectability (A), dynamic sweep frequency rheological studies (B), and scanning electron microscope (SEM) images (C) of Gelma and Gelma with Mn-HANWs.

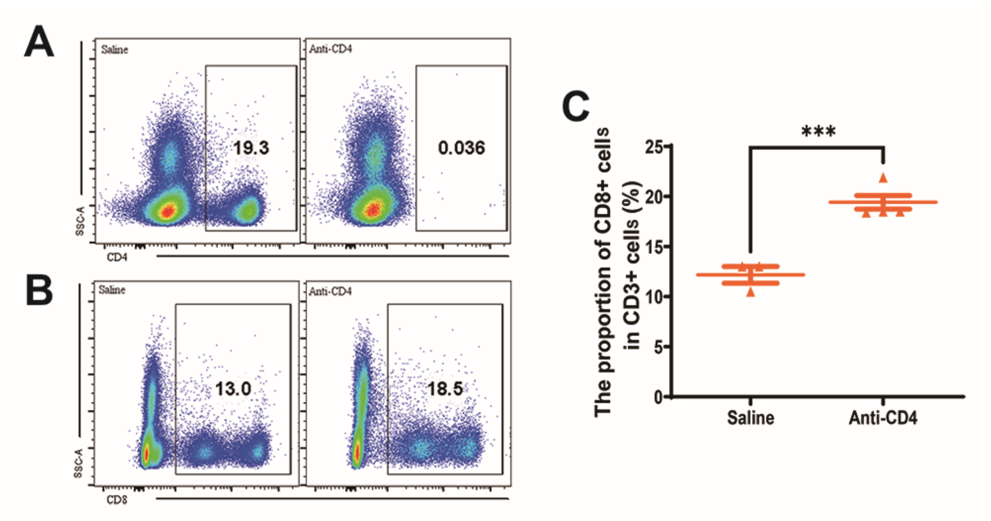

Figure S3. A) The proportions of $CD4^+$ and $CD8^+$ T cells in the $CD45^+CD3^+$ blood cells of mice treated with saline or anti-CD4 antibodies. B) The quantification of $CD8^+$ T cells in the $CD45^+CD3^+$ blood of mice treated with saline or anti-CD4 antibodies. N≥3, **P* < 0.05, ***P* < 0.01, ****P* < 0.001.

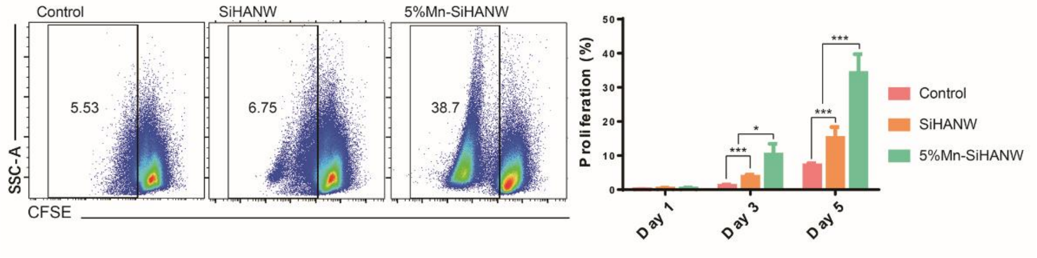

Figure S4. $CD4^+$ T cell proliferation stimulated by PBS (Control), SiHANW, and 5%Mn-SiHANW. **P* < 0.05, ***P* < 0.01, ****P* < 0.001.

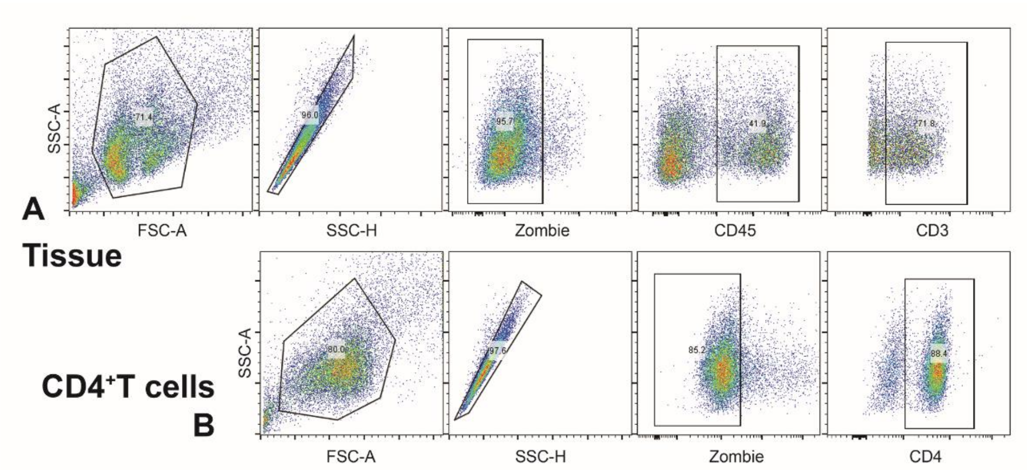

Figure S5. The gating strategy of flow cytometry analyzing tissue (A) and CD4^{+} T cell (B) samples.
